# Mapping the moral architecture of effective and extraordinary altruism

**DOI:** 10.1093/pnasnexus/pgaf326

**Published:** 2025-10-22

**Authors:** Kyle Fiore Law, Stylianos Syropoulos, Paige Amormino, Abigail Marsh, Liane Young, Brendan Bo O’Connor

**Affiliations:** Global Futures School of Sustainability, Arizona State University, Tempe, AZ 85287, USA; Global Futures School of Sustainability, Arizona State University, Tempe, AZ 85287, USA; Department of Psychology, Georgetown University, Washington, DC 20007, USA; Department of Psychology, Georgetown University, Washington, DC 20007, USA; Interdisciplinary Neuroscience Program, Georgetown University, Washington, DC 20007, USA; Department of Psychology & Neuroscience, Boston College, Chestnut Hill, MA 02467, USA; The Schiller Institute for Integrated Science and Society, Boston College, Chestnut Hill, MA 02467, USA; Department of Psychology, University at Albany, State University of New York, Albany, NY 12222, USA

**Keywords:** morality, prosocial behavior, effective altruism, extraordinary altruism, moral foundations

## Abstract

While global challenges demand both equitable and effective altruism, people often prioritize those nearby and overlook the impact of their prosocial actions. Here, we examined what moral values support altruism that is both equitable (helping others impartially regardless of proximity or group membership) and effective (helping impactfully by maximizing welfare gains). We studied two rare populations: effective altruists (EAs; *N* = 119), defined by their philosophical commitment to impartiality and maximizing impact, and extraordinary altruists (XAs; *N* = 65), defined by their real-world decision to donate an organ to a stranger—an act of radical impartiality, though not explicitly impact-maximizing. A demographically similar general population sample (*N* = 176) served as a comparison group. Participants completed moral measures from pluralistic theories (i.e. Moral Foundations Questionnaire, Morality-as-Cooperation Questionnaire [MAC-Q]), along with the Moral Expansiveness Scale (MES), and the Oxford Utilitarianism Scale (OUS). They also completed assessments of equitable, effective, and combined altruistic orientations. Both altruistic groups showed elevated moral expansiveness (MES) and impartial beneficence (OUS) relative to controls. EAs, compared to XAs and controls, more strongly endorsed utilitarian principles (including instrumental harm on the OUS) and deprioritized traditional cooperative values (e.g. reciprocity, deference on the MAC-Q). Familial loyalty (MAC-Q) negatively predicted altruistic orientations across groups. Surprisingly, group loyalty—conceptualized as parochial by pluralistic moral theories—positively predicted equitable and effective altruism across groups, challenging assumptions that parochial values necessarily constrain prosociality. These findings instead suggest inclusive forms of loyalty may help promote altruism that is both equitable and effective.

Significance Statement This investigation finds that individuals who engage in effective and extraordinary altruism—respectively, helping strangers through high-impact giving or nondirected living organ donation—prioritize moral concern for a broad range of others and endorse impartial beneficence more strongly than typical adults. However, effective altruists differ from extraordinary altruists, *deprioritizing* traditional cooperative values, like deference to authority and reciprocity, while *prioritizing* instrumental harm more—harming the few to benefit the many. Surprisingly, group loyalty predicts more equitable and effective altruism across all three samples, challenging longstanding views that loyalty constrains altruistic impartiality. These findings suggest that both expansive and even traditionally parochial values can support equitable, impact-maximizing altruism when expanded to encompass broader moral communities.

## Introduction

Human morality is widely believed to have evolved to promote cooperation within small, close-knit groups (1–6). However, both social psychologists (7–12) and moral philosophers (13–17) have long argued that moral concern for the well-being of others can—and should—extend beyond parochial boundaries. Indeed, some philosophical and theoretical perspectives suggest that the “moral circle”—the array of entities people deem worthy of “moral concern,” or protection from harm and suffering—has expanded over time, with concern increasingly broadening to include those who are socially, geographically, or even biologically distant (i.e. a concept referred to as “moral expansiveness”) (13, 14, 18). This tension between morality as an evolved mechanism for local cooperation and as a foundation for a more expansive circle of concern lies at the heart of understanding both the psychological capacities that enable “equitable” or “impartial” altruism (i.e. defined here as helping others regardless of proximity or group membership) and the boundaries that may constrain human prosociality (3, 19). On the one hand, values like care and fairness can push moral boundaries outward; on the other, values like loyalty are thought to anchor them to the ingroup (20–25). As a result, while humans are capable of extraordinary generosity, everyday helping behavior tends to favor those who are close, familiar, and similar (12, 26–28). People often prioritize local or known others, even judging aid to distant strangers as morally wrong when it comes at the expense of closer beneficiaries (26, 29–33).

Yet, some individuals defy this pattern. Exceptionally altruistic people provide a rare glimpse into the values that support altruism beyond conventional boundaries. Prior research (22, 23) has already begun to illuminate the moral architecture of extraordinary altruists (XAs), such as nondirected, living organ donors, who make profound personal sacrifices to help strangers they will never meet (34–37). This work has shed light on how the minds of such altruists are characterized by moral orientations toward “impartial beneficence” (i.e. the moral principle that one ought to promote the greatest benefit wherever it is needed most, without regard for proximity, personal identity, or group affiliation (38)) and harm aversion. Still, the picture is incomplete. Many of the world's most pressing challenges, including poverty, climate injustice, and global health disparities, require not just *equitable* altruism—nor the mere endorsement of abstract principles like moral expansiveness or impartial beneficence (IB)—but also “effective” or “impact-maximizing” altruism (i.e. defined here as striving, whenever helping others, to reduce the greatest amount of suffering per unit of sacrifice) (17, 39). In this regard, effective altruists (EAs), individuals explicitly committed to doing the most good by directing resources to high-impact causes (i.e. those that save the most lives per dollar), offer critical and complementary insight (16, 39–42).

Both groups exemplify altruism that transcends parochial boundaries but differ in definition (43, 44). XAs are defined by a real-world equitable act (i.e. donating a kidney to a stranger) (34–37), while EAs are defined by a philosophical commitment to helping equitably and effectively, often via data-driven giving to high-impact causes that help distant or statistically represented lives (16, 39–42). Despite these differences, both groups show overlap in how they behave (44). EAs explicitly endorse equity and effectiveness in both practice and laboratory tasks, and XAs, though not expressly committed to equitability or effectiveness on an *ideological* level, also display impact-oriented prosociality in experimental settings (43).

Here, we compare the moral architectures of EAs and XAs to demographically matched controls to test whether distinct moral values support altruism that is both impartial (equitable) and impact-maximizing (effective), an important combination in today's world (17, 39). By linking moral values to laboratory tasks and behavioral measures of equitable and effective prosociality, we assess how the values underlying these forms of altruism differ across: (i) EAs, who explicitly endorse both equitability and effectiveness through dogmatic affiliation with the EA movement and associated giving; (ii) XAs, defined by a real-world equitable act rather than ideological alignment, yet who also show preferences for effectiveness in laboratory tasks (43); and (iii) ordinary adults, who are not aligned with either form of altruism but, as the majority, hold the greatest potential to scale these values. This work provides empirical and theoretical insight into a moral architecture that reflects a longstanding philosophical vision of expanding care and concern *equitably* across space, social group, and species (13, 14, 18), and one well-suited for *effectively* addressing today's most urgent societal challenges (17, 39).

### Applying a special population approach to gain insight into the moral architecture of equitable and effective altruism

Prominent theories of “moral pluralism” (i.e. the view that morality consists of multiple, coexisting moral values rather than a single core principle), such as Moral Foundations Theory (19, 45) (MFT; see Haidt and Graham (46)) and, more recently, Morality as Cooperation Theory (2, 3) (MAC; see Curry (3))^a^, propose that the moral value of loyalty evolved to preserve group solidarity. Regarding loyalty, Haidt and Graham write about the evolution of “special social-cognitive abilities backed up by strong social emotions related to recognizing, trusting, and cooperating with members of one's co-residing ingroup while being wary and distrustful of members of other groups (46).” This “binding” moral value is thought to scaffold parochial altruism: cooperative behavior directed primarily toward close others and ingroup members (21, 48–50). Yet, research from the same pluralist traditions finds that people also endorse more universalist moral values, such as care (47, 51) and fairness, which extend moral obligations beyond one's immediate social circle (21, 25, 52–54). Graham and colleagues write, “The “individualizing” concerns of maximizing care, fairness, and justice act as primarily centrifugal forces, expanding moral regard outward without prejudice (21).” These “individualizing” values—so named because they prioritize the rights of individuals based on their level of need over group cohesion—nonetheless tend to be applied unevenly.

People typically display empathy gaps (7, 8, 30, 55) and altruistic biases (6, 26, 42) that favor socially and physically proximate beneficiaries—those who are similar, familiar, or geographically near. Moreover, people often fail to prioritize consequentialist considerations in their moral decision-making (56–58), overlooking how actions might maximize welfare or mitigate suffering, especially when the beneficiaries are distant or abstract. This presents a challenge: many of the world's most pressing problems require impact-maximizing prosociality that transcends conventional boundaries of identity and proximity (16–18, 39, 40). Because such equitable and effective altruism is rare, individuals who regularly act on these values, like EAs (16, 31, 39) and extraordinary altruists (35–37) (XAs), offer a unique opportunity to understand what enables people to help others impartially and impactfully. However, the moral psychology of such altruism remains poorly understood. Do EAs and XAs prioritize moral values differently from typical adults? Is the binding moral value of loyalty, which supports group cohesion (2, 21), deprioritized in favor of impartial values like care, fairness, and moral expansiveness among these populations (25, 52)? Do these individuals from both of these populations more strongly endorse IB (38)? And, while both XAs and EAs embody altruism that extends beyond parochial boundaries, might they differ in their underlying moral commitments, with XAs aligning more with expansive moral concern for individuals in need, and EAs more with outcome-focused principles rooted in utilitarianism, including a greater willingness to accept the dark side of utilitarianism—sacrificing the few for the benefit of many (38)?

Whereas much prior research has focused on how moral values support prosociality within typical populations, few empirical studies have examined how these values operate in exceptionally altruistic individuals. Recent work has begun to map the moral architecture of extraordinary altruists, including both nondirected organ donors (22) and individuals who donate a substantial proportion of their income to charity (23). Findings from altruistic kidney donors suggest these individuals show heightened concern for harm and endorsement of IB relative to controls, without increased acceptance of instrumental harm or significant differences in other basic values from pluralistic moral theories like MFT (e.g. fairness, loyalty, authority, purity) (22). Similarly, individuals who make large, sustained financial commitments to charitable giving have been found to exhibit greater moral expansiveness and IB than controls, with some evidence of increased endorsement of instrumental harm (23). Finally, although not focused specifically on moral values, recent research has found that extraordinary altruists (e.g. organ donors) and self-identified EAs differ in their underlying cognitive and emotional profiles: While both EAs and XAs tend to score higher on measures capturing equitable and effective prosociality, XAs tend to exhibit heightened empathic capacity, whereas EAs show greater emphasis on deliberative, rational processing (43).

Taken together, the existing literature hints that different forms of exceptional altruism may reflect distinct moral profiles: one centered on expansive moral concern and aversion to harm, and another that also incorporates elements of outcome-focused reasoning. To date, however, no study has systematically examined the moral architecture of individuals who explicitly identify as EAs, nor compared them directly to those of extraordinary altruists and general population controls. Moreover, no research to date has systematically linked the moral values of different types of exceptional altruists (EAs nor XAs) to the specific attitudes, judgments, and behaviors that characterize distinct modalities of prosociality, such as efforts to help others impartially (equitable or impartial altruism) versus outcome-oriented efforts that maximize impact (effective or impactful altruism).

#### The current research

The current study addresses these gaps by assessing differences in moral values and beliefs across three populations: EAs, Extraordinary Altruists (XAs; in this case, the rare population of nondirected living organ donors), and general population controls. We examine how these values relate to key forms of prosociality in each of the three groups, including: (i) Equitable or Impartial altruism: helping others regardless of distance or similarity, (ii) Effective or Impactful altruism: prioritizing causes that maximize welfare gains, (iii) Equitable and effective altruism: prioritizing distant causes that maximize welfare gains over closer albeit less-impactful causes, and (iv) Real-world charitable action: self-reported levels of general charitable giving and volunteerism, irrespective of whether such acts are equitable or effective. By comparing these groups across multiple moral domains, including moral expansiveness (the breadth and depth of individuals’ moral concern for the welfare of others), individualizing values (care, fairness), binding values (loyalty in particular, but also authority and purity), and utilitarian principles (IB, instrumental harm), we provide a comprehensive examination of the moral architecture underlying both everyday and exceptional altruism. In doing so, this research advances our understanding of how prosocial behavior is shaped, bounded, and expressed as a function of the moral values people hold, and highlights the values that support not only ingroup cooperation, but also equitable altruism and impact-driven altruism across spans of distance. Table S1 in the Supplementary Online Materials presents key demographic information for each sample and highlights the key differences between the two groups of exceptional altruists compared here.

## Results

### Population differences in moral beliefs and values

**Fig. 1.**
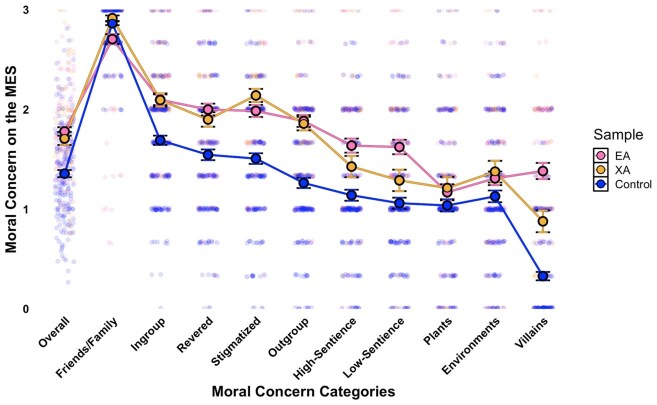
Overall and finer-grained distinctions in moral concern between EAs, extraordinary altruists, and general population controls. Note. Jitter density plots with means and 95% CI illustrating moral concern across different groups on the Moral Expansiveness Scale (MES), including friends and family, ingroup members, revered figures, stigmatized and outgroup humans, high- and low-sentience animals, plants, environments, and villains. The overall moral circle size is also shown. Different colors represent scores for EAs, extraordinary altruists (XAs), and demographically matched controls.

**Fig. 2.**
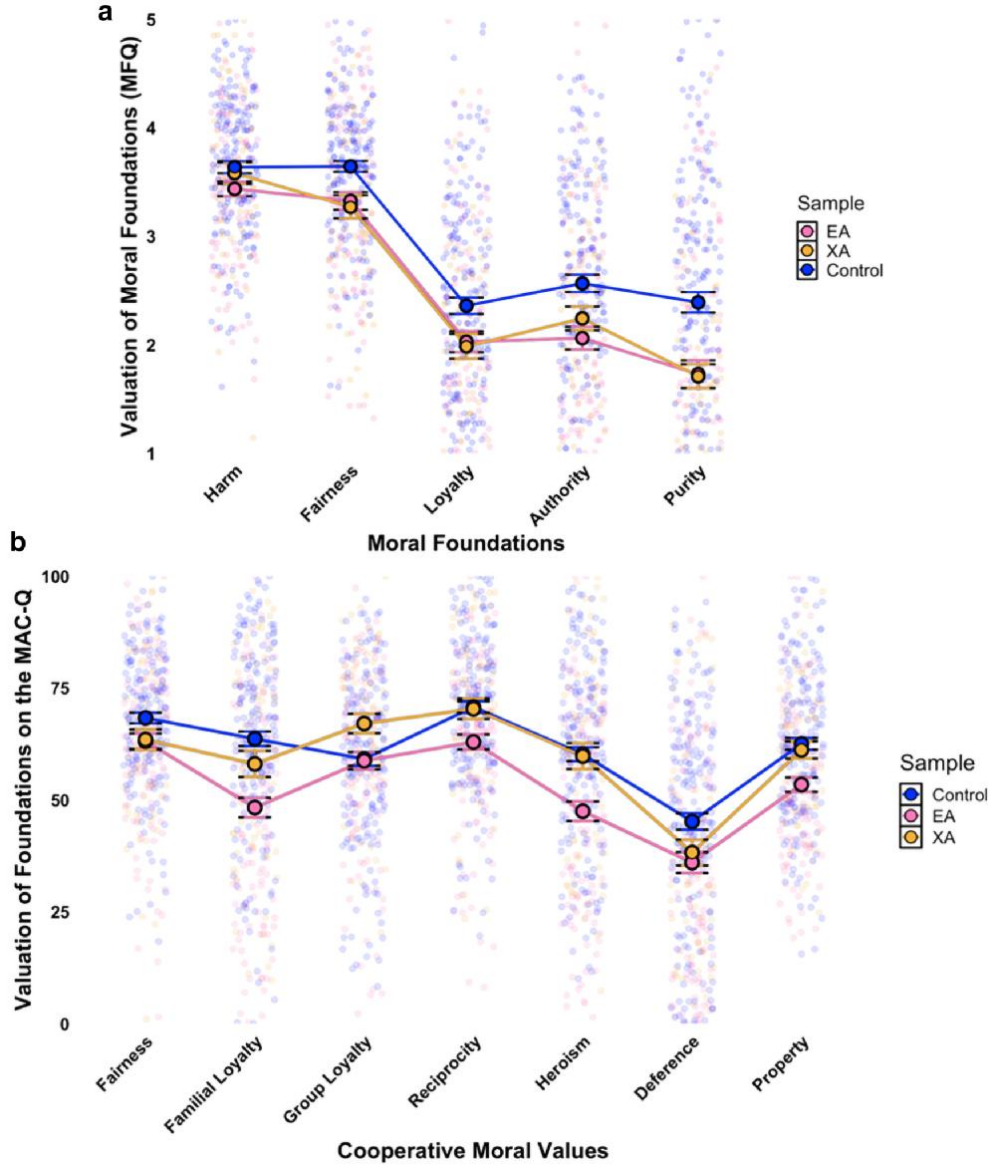
Moral values on the MFQ and MAC-Q between EAs, extraordinary altruists and general population controls. Note. Jitter density plots with means and 95% CI illustrating moral valuation across different moral domains. a) Scores from the Moral Foundations Questionnaire (MFQ) for the prosocial foundations of harm and fairness, the parochial foundation of loyalty, and, for exploratory purposes, the foundations of authority and purity. b) Scores from the Morality as Cooperation Questionnaire (MAC-Q) for the prosocial value of fairness, the parochial values of familial and group loyalty, and, for exploratory purposes, the values of reciprocity, deference to authority, heroism, and property rights. Different colors represent scores for EAs, extraordinary altruists (XAs), and demographically matched controls.

**Fig. 3.**
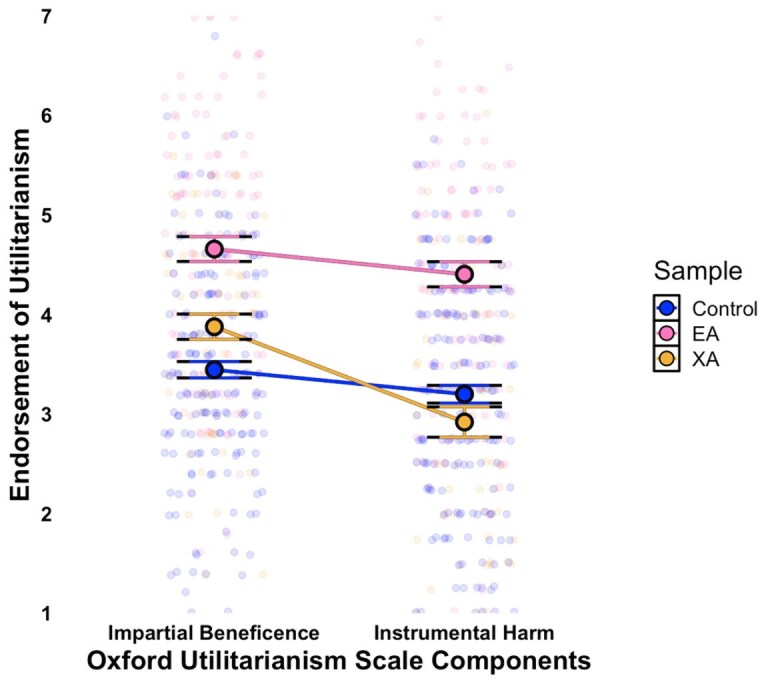
Endorsement of the utilitarian values of IB and instrumental harm between EAs, extraordinary altruists, and general population controls. Note. Jitter density plots with means and 95% CI illustrating moral endorsement on the Oxford Utilitarianism Scale (OUS). Scores reflect IB, representing a commitment to maximizing overall well-being regardless of personal or relational ties, and Instrumental Harm (IH), representing the acceptance of causing harm for the greater good. Different colors represent scores for EAs, XAs, and demographically matched controls.

We first assessed how the three samples differed on the measures of moral beliefs and values (see Table S2 for details pertaining to each measure). We hypothesized that members of the altruistic populations, when compared to general population controls, would ascribe greater moral prioritization to the “individualizing” values of care and fairness on the MFQ and fairness on the MAC-Q, as well as the utilitarian principle of IB on the OUS, values that existing theory and evidence suggest are associated with prosociality in general and prosociality across group boundaries in particular (20, 21, 59, 60). Similarly, we hypothesized that altruists would manifest more expansive moral circles, ascribing greater moral concern to a greater number of entities spanning a range of distance from oneself (23, 24). Conversely, we hypothesized that members of the altruistic populations, compared to general population controls, would ascribe less moral prioritization to the “binding” values of loyalty on the MFQ, and familial and group loyalty on the MACQ, values which typically promote cooperation within groups, but at times can instead impede cooperation between them (2, 20, 21). Our hypotheses were partially supported (Table S3 and Figures 1–3 provide inferential statistics and visualizations of the observed patterns, respectively).

#### Prosocial moral values

As predicted, both EAs and XAs scored higher than controls on overall moral concern (Moral Expansiveness Scale; MES) and IB (from the Oxford Utilitarianism Scale; OUS), with EAs showing the highest IB scores. These findings replicate prior work showing elevated IB among organ donors (22) and Giving What We Can Pledgees (23)—another group of extraordinary altruists committed to donating at least 10% of lifetime income—who also exhibit more expansive moral circles and stronger IB endorsement. Crucially, this is the first empirical demonstration of these patterns among self-identified EAs, who are defined not only by impartial concern but also by a commitment to maximizing impact. These results highlight the central role of a broad moral circle in shaping the values of those pursuing both equity and effectiveness in their altruism. Moreover, the especially high endorsement of IB among EAs lends credence to a broader view of utilitarian ethics grounded in compassionate, impartial concern for others, rather than defined solely by its more controversial association with instrumental harm (38, 59, 61).

Yet, intriguingly, and contrary to both our predictions and prior research among organ donors (22), both EAs and XAs scored lower than controls on fairness (MFQ), with EAs also scoring lower on fairness as measured by the MAC-Q. Additionally, no group differences emerged for harm/care (MFQ), diverging from pre-registered predictions and past findings of modest elevation among organ donors (22). While null results for harm/care may reflect its broad consensus (most people agree harm is wrong and care is good (20)), the fairness findings are more complex. Fairness intuitively aligns with the equitable behavior observed among altruists (43), raising the possibility that these patterns reflect measurement limitations. For instance, both the MFQ and MAC-Q conflate fairness-as-equitability (equal shares) with fairness-as-proportionality (reward-by-contribution), which may obscure true group differences. Newer tools better disentangle these dimensions (e.g. Atari et al (60).), and future work should examine whether exceptionally altruistic populations score higher on equitability alone. Endorsing equitability but not proportionality would align with these populations’ commitment to impartial altruism, reflected in their tendency to help others regardless of traditional standards of merit, contribution, or deservingness.

#### Parochial moral values

In line with our predictions, both EAs and XAs scored lower than controls on the moral foundation of loyalty (MFQ), suggesting a reduced emphasis on preferential commitment to close others. Additionally, EAs scored lower than controls (and XAs) on familial loyalty (MAC-Q). Surprisingly, however, XAs scored higher on group loyalty on the MAC-Q compared to both EAs and controls. This finding is striking given that leading models of cooperation characterize loyalty as a double-edged sword that enhances cooperation within groups while inhibiting cooperation between them (21, 62). The fact that XAs, who undergo great personal sacrifice to help complete strangers, still exhibit a strong sense of loyalty to their group members suggests that loyalty may not necessarily compete with efforts to promote the greater good. Instead, and in alignment with emerging insights suggesting altruistic organ donors maintain fulfilling close social relationships (63), it appears that a deep moral commitment to those who are closest can coexist with a broader altruistic concern for distant strangers.

#### Finer-grained distinctions in the moral circle

To better understand why altruists who help strangers may also endorse strong group loyalty, we also examined *to whom* they assign moral concern (see Figure 1 for visualization and Table S4 in the Supplementary Online Materials for inferential statistics). Most people prioritize close others in their moral circles (24, 25, 64, 65), but altruists may extend concern more broadly. Thus, we explored not just whether altruists have greater *scope* but also greater *depth* in their moral circles than controls. If altruists include more distant others in their moral community, and assign them greater moral weight, their loyalty may reflect an inclusive moral group rather than narrow ingroup favoritism.

In terms of moral concern for the closest others, XAs did not differ significantly from controls, while EAs expressed even less concern for friends and family than both groups. Yet across all other human categories—ingroup, revered, stigmatized, outgroup, and villain—both altruistic groups showed greater concern than controls. The same held for high-sentience animals, with EAs also extending more concern to low-sentience animals. No group differences emerged for plants or natural environments. These findings may clarify why XAs show elevated loyalty despite helping strangers. Their moral concern extends broadly across human targets, not just close others. This suggests that group-based commitments can coexist with equitable altruism, especially when the moral “ingroup” is defined inclusively. In contrast, EAs’ comparatively lower concern for close others may help explain why their group loyalty is not similarly heightened.

That said, this is only one potential explanation. Heightened group loyalty among XAs may reflect not just a broader moral circle but a more general intensification of moral commitments, including to conventional groups like one’ immediate community. Rather than competing with equitable and effective altruism, such loyalty could serve as a motivational base, helping moral concern extend outward. In this view, loyalty may not limit prosocial action but instead act as a springboard for it.

#### Sample differences on support for the dark side of utilitarianism

For exploratory purposes, we also examined whether EAs showed greater endorsement of Instrumental Harm (IH) on the OUS relative to XAs and controls. IH refers to the idea that it is morally acceptable to cause harm to a few people if doing so produces a greater good for many. Prominent EA figures have asserted that the Effective Altruism philosophy aligns with utilitarian ethics primarily through IB, which prioritizes maximizing welfare in prosocial contexts (16, 39). While foundational EA texts explicitly reject IH, research has never tested whether EA members endorse this principle empirically. Strikingly, EAs not only endorse IH, but score 0.96 and 1.18 standard deviations higher on average than controls and XAs, respectively.

#### Exploratory evaluations of sample differences in moral foundations and cooperative moral values

Although we did not have pre-registered predictions, we explored how the groups differed on remaining moral values from the pluralistic theories (i.e. MFQ: authority, purity; MAC-Q: reciprocity, deference to authority, heroism, property rights). Results revealed notable differences. EAs assigned significantly less moral value than controls to all six domains, while XAs differed from controls only on purity. EAs also valued reciprocity, heroism, and property rights significantly less than XAs, highlighting their distinct moral profiles.

These differences suggest that EAs deprioritize traditional moral values related to hierarchy/social cohesion (authority, deference, purity), mutual obligation (reciprocity), and ownership (property rights), embracing a more impartial and universalist moral framework that stands in contrast to the expectations of biological models of cooperation, which emphasize the evolutionary advantages of in-group loyalty, reciprocity, and social cohesion (2, 3, 66, 67). Their lower valuation of heroism may reflect a preference for structural, large-scale impact over individual sacrifice (16, 39, 40). In contrast, XAs’ moral values align more closely with the general population, suggesting that while they extend moral concern broadly, they do not reject conventional cooperative norms. This distinction may further help explain why XAs—but not EAs—exhibit heightened group loyalty, as they maintain stronger ties to social and reciprocal obligations despite morally valuing distant others and engaging in costly altruism benefitting strangers.

### The moral architecture of equitable and effective altruism

After identifying differences in moral values across the three samples, we examined which values predict prosociality across various laboratory metrics and behavioral tasks. Details pertaining to these tasks are outlined in Table S2. In short, some measures assessed overall prosociality, such as self-reported annual time volunteering and money donated to charitable causes. Others captured distinct aspects of altruism, including equity (e.g. helping others regardless of distance or similarity), effectiveness (e.g. prioritizing causes that maximize welfare gains), or both (e.g. choosing to help distant beneficiaries when doing so yields the greatest welfare impact). An important caveat is that two outcome measures—the Social Discounting Task (SDT) and the Behavioral Donation Task (BDT)—served as behavioral proxies involving dichotomous resource-allocation decisions. Though both were low-stakes and imposed no personal cost, only the BDT was incentive-compatible: one randomly selected trial per subject determined a real $1 donation. Thus, while the SDT involved hypothetical tradeoffs, the BDT involved modest real-world consequences.

Similar to our hypotheses for sample differences in moral values, we hypothesized that scores on the measures capturing moral facets theoretically aligned with expansive prosociality (i.e. MFQ: harm, fairness; MAC-Q: fairness; OUS: IB; overall moral concern on the MES) would correlate positively with scores on the prosociality measures. Conversely, we hypothesized that scores on the measures of moral facets theoretically aligned with parochialism (i.e. MFQ: Loyalty; MAC-Q: Familial Loyalty, Group Loyalty) would associate negatively with scores on the prosociality measures.

To test these hypotheses, we conducted separate linear regression models for each category of moral predictors: (1) the Moral Expansiveness Scale (MES), assessing overall moral concern; (2) the Moral Foundations Questionnaire (MFQ), including all five foundations as simultaneous predictors to examine the unique effects of harm, fairness, and loyalty, while controlling for each other as well as the exploratory values of authority and purity; (3) the Morality-as-Cooperation Questionnaire (MAC-Q), with all seven subscales entered simultaneously to assess the predictive roles of fairness, familial loyalty, and group loyalty, while accounting for each other as well as the exploratory values of reciprocity, deference, heroism, and property rights; and (4) the Oxford Utilitarianism Scale (OUS), including both components—IB and instrumental harm—to isolate the predicted effects of IB while controlling for the endorsement of harm-based trade-offs (which was measured for exploratory purposes). Because associations with the exploratory moral values were less consistent across samples (and were not part of our pre-registered hypotheses) we do not interpret these relationships directly. Nonetheless, full results from these models, including associations with the exploratory values, are presented in Table S5 (for linear regressions) and Table S6 (for bivariate relationships).

#### Effective altruists

Mirroring the sample differences in moral values presented above, among EAs, the most consistent predictors of altruistic attitudes, judgments and behaviors were impartial beneficence on the OUS and overall moral concern on the MES, especially for attitudes and judgments regarding equitable, high-impact (i.e. effective) helping. As hypothesized, IB predicted stronger endorsement of both equitable and effective altruistic attitudes on the Effective Altruism Interest Scale (i.e. the Expansive Altruism [EAIS-EX] and Effectiveness Focus [EAIS-EF] subscales), as well as higher scores on the Moral Judgment Vignettes (MJV) and SDT (i.e. measures that reflect moral judgments and decisions prioritizing altruism that is both equitable in scope and maximized in impact). However, IB did not predict greater monetary donations to effective causes on the BDT, for which participants completed 16 forced-choice trials deciding whether to donate money to a high-impact cause (e.g. saving lives) versus a lower-impact one (e.g. improving lives). This suggests that principled endorsement of impartiality does not necessarily translate into altruistic behavior involving real financial trade-offs among EAs.

Yet, moral concern on the MES predicted scores on the EAIS-EX, MJV, and SDT, *and* showed a significant positive relationship with donations to effective causes on the BDT, suggesting that a more expansive moral circle supports not only more equitable, but also impact-maximizing altruistic engagement in the context of both attitudes *and* behaviors. In other words, extending moral concern more broadly across varying levels of target distance and similarity (i.e. greater moral expansiveness) predicts greater support for consequentialist prosocial outcomes, even when such outcomes require trading off less-effective aid to those traditionally prioritized or held in higher moral regard. Intriguingly, however, neither impartial beneficence nor moral concern as measured by the MES was associated with self-reported engagement in Real World Charitable Action (RWCA), including the percentage of time and money participants reported volunteering and donating. This suggests that more expansive moral values may be more predictive of how EAs *prioritize* altruism than *how frequently* they engage in it overall.

Although no sample differences were observed for the moral value of harm reduction on the MFQ, in line with our predictions, it was a strong predictor of altruistic outcomes among EAs, supporting its foundational role in prosocial motivation (19, 47). Among EAs, valuation of harm reduction predicted higher scores on the same outcomes as moral concern on the MES (i.e. scores on the EAIS-EF, EAIS-EX, MJV, BDT, and SDT). These associations emphasize the continued relevance of compassion in altruism that prioritizes both equity and effectiveness (21, 25, 64). The value of fairness, however, yielded mixed results. MFQ fairness did not significantly predict any altruistic outcomes, though it trended positively with reported levels of volunteerism (RWCA-Time; *β* = 0.19, 95% CI [−0.03, 0.41], *P* = 0.088), and trended negatively with scores on the SDT (*β* = 0.16, 95% CI [−0.46, 0.02], *P* = 0.078). Meanwhile, MAC-Q fairness significantly predicted scores on both the EAIS-EX and MJV. These findings, which parallel the observed population-level differences in fairness valuation, suggest that fairness does not consistently relate to altruistic outcomes—at least not as measured here, where existing instruments may conflate the distinct principles of equitability and proportionality (60).

As predicted, familial loyalty was negatively associated with scores on the EAIS-EX, EAIS-EF, BDT, and SDT, supporting the idea that prioritizing close kin may conflict with more impartial forms of giving (1, 21, 29, 31). Similarly, loyalty on the MFQ showed negative associations with scores on the MJV and BDT. Yet, somewhat unexpectedly, though in alignment with the heightened levels of group loyalty among XAs, group loyalty on the MAC-Q predicted greater prioritization of both altruistic equity and altruistic effectiveness (scores on the EAIS-EX, EAIS-EF, and SDT) among EAs, suggesting that in some cases, loyalty, at least when directed beyond kin and towards the broader groups to which people belong, may serve a more faciliatory role in motivating expansive prosocial concern than existing theoretical accounts would suggest.

#### Extraordinary altruists

Owing to the rarity of living nondirected organ donors, it is worth noting that we were not powered within the sample of extraordinary altruists (XAs) to detect small effect sizes. As such, we report trends toward significance below but urge caution in interpretation, emphasizing effect sizes (i.e. standardized regression coefficients, “*β*”) and confidence intervals over *P*-values for these relationships. Nonetheless, even given these constraints, we observed some intriguing associations that largely corresponded with those observed among EAs. In line with our predictions, among XAs, impartial beneficence predicted attitudes aligned with altruistic equity on the EAIS-EX, again supporting the role of impartial concern in shaping judgments about helping others regardless of their distance. As with EAs, impartial beneficence did not predict donations on the BDT. However, in addition to also predicting higher scores on the EAIS-EX, moral expansiveness trended towards significance in predicting a greater proportion of donations to effective causes on the BDT (*β* = 0.23, 95% CI [−0.01, 0.48], *P* = 0.061), suggesting that for XAs and EAs alike, expansive moral concern may be more behaviorally expressed and related not only to more equitable altruism, but effective altruism as well.

In contrast to our predictions, neither harm nor fairness significantly predicted altruistic outcomes among XAs. This may reflect the unique moral profile of this group, whose real-world altruism is already expressed at extreme levels (43), potentially independent of abstract moral principles. In a similar manner to the observations among EAs, however, the role of loyalty was nuanced among the XA sample as well. In directional alignment with our predictions, loyalty on the MFQ trended negatively with scores on the BDT (*β* = −0.36, 95% CI [−0.76, 0.04], *P* = 0.077), while familial loyalty on the MAC-Q trended negatively with reported levels of volunteerism (RWCA-Time; *β* = −0.37, 95% CI [−0.80, 0.07], *P* = 0.095). Yet, group loyalty significantly predicted more equitable altruistic attitudes on the EAIS-EX and trended positively with effective donation behaviors on the BDT (*β* = 0.37, 95% CI [−0.00, 0.74], *P* = 0.052), further suggesting that loyalty—when extended beyond narrow kinship boundaries—may complement, rather than compete with, altruistic equity and impact.

#### General population controls

Among controls, patterns were weaker overall but still aligned with theoretical expectations in most instances. Impartial beneficence positively predicted both equitable (EAIS-EX) and effective (EAIS-EX) altruistic attitudes and behaviors (scores on the SDT), suggesting that impartial concern for the well-being of others promotes both equitable and effective altruistic orientations even among typical adults. Similarly, more expansive moral circles were associated with more equitable altruistic attitudes (EAIS-EX) and tendencies to prefer larger rewards for beneficiaries regardless of their distance on the SDT, while they trended positively with reported monetary charitable contributions (RWCA-Money; *β* = 0.14, 95% CI [−0.01, 0.29], *P* = 0.070).

Intriguingly, fairness showed greater alignment with theoretically predicted patterns among controls than among the altruistic samples. Specifically, MFQ fairness trended positively with effective altruistic attitudes on the EAIS-EF (*β* = 0.17, 95% CI [−0.00, 0.34], *P* = 0.056) and significantly predicted both a greater proportion of effective donation decisions on the BDT and more favorable moral judgments of equitable and effective altruism on the MJV. Similarly, MAC-Q fairness was positively associated with scores on the BDT, EAIS-EX, and MJV. The differences between the predictive power of fairness between altruists and controls may be explained, in part, by the fact that effective and extraordinary altruists tend to place lower value on fairness overall, as shown in earlier group comparisons. As a result, fairness, at least according to its operationalization in the present investigation, may play a less central motivational role in guiding their altruistic behavior.

While the value of harm reduction on the MFQ, as predicted, was positively associated with equitable altruistic attitudes, and trended positively with reported monetary charitable contributions (RWCA-Money; *β* = 0.16, 95% CI [−0.02, 0.35], *P* = 0.084), it showed a trending negative association with effective altruistic attitudes (EAIS-EF) among controls (*β* = −0.18, 95% CI [−0.36, 0.01], *P* = 0.059). Though this finding should be interpreted with caution, one possible explanation is that typical adults tend to perceive harm to be more morally pressing when it affects those who are socially or emotionally closer (7, 21, 25, 31, 68). As a result, typical adults who are more harm averse and “feel” the impact of harm more strongly for close others, may be less supportive of the notion that altruistic impact is more ethically important than social ties.

**Fig. 4.**
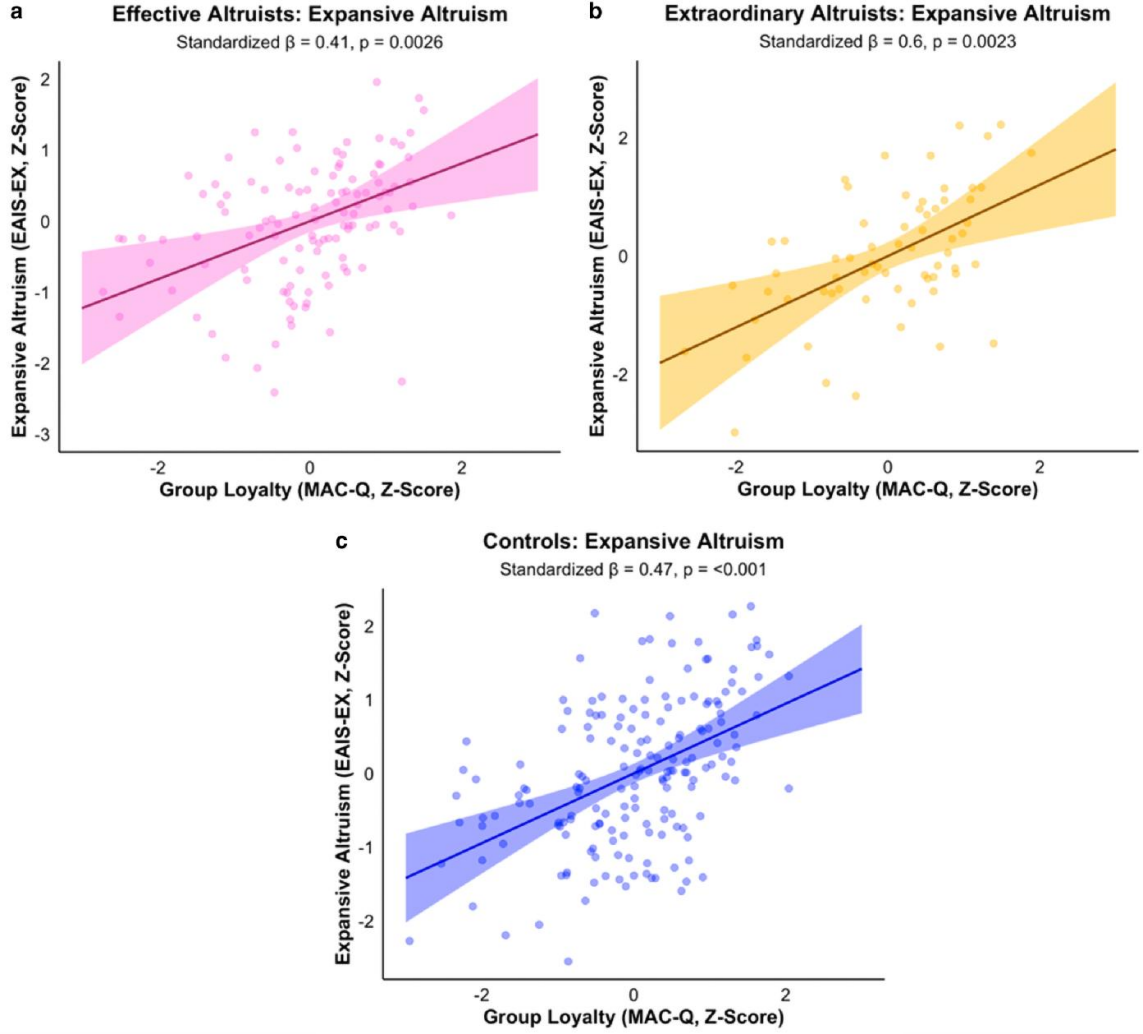
Relationships between group loyalty on the MAC-Q with expansive altruism on the EAIS (altruistic equity) among EAs, XAs, and general population controls. Note. Standardized regression lines and 95% CI illustrating the relationship between Group Loyalty (MAC-Q) and scores on the EAIS-EX—which captures attitudes aligned with equitable/impartial altruism—among EAs, extraordinary altruists (XAs), and demographically similar controls. Points represent partial residuals after accounting for all other cooperative moral values on the MAC-Q (MAC-Q subscales: Fairness, Familial Loyalty, Reciprocity, Heroism, Deference, Property Rights). Regression lines and associated confidence bands depict the unique effect of Group Loyalty on the outcome within each sample group, holding all other MAC-Q foundations constant. Panels are faceted by sample group (a–c). Though only the relationships with the EAIS-EX are displayed here, Group Loyalty was a significant, positive predictor of numerous facets of equitable (impartial) and effective (impactful) altruistic orientations across sample groups (see Table S5).

Finally, with respect to loyalty, the findings among controls were similarly surprising to those observed in the altruistic populations. While familial loyalty, as predicted, was negatively associated with moral judgments of equitable and effective altruism (MJV), group loyalty on the MAC-Q positively predicted equitable altruistic attitudes (EAIS-EX), effective altruistic behaviors (BDT), and trended positively with moral judgments of effective altruism directed towards distant others (MJV; *β* = 0.18, 95% CI [−0.03, 0.39], *P* = 0.092). Likewise, loyalty on the MFQ—a more general measure of allegiance to one's ingroup—was positively associated with effective altruistic attitudes (EAIS-EF), diverging from expectations and contrasting with the generally negative or null associations observed in the altruistic samples. These patterns across the three samples suggest that ingroup-oriented (“binding”) values do not universally inhibit impartial altruism. Instead, they point to a more nuanced picture: when loyalty is narrowly directed, such as toward close kin, it may conflict with altruism that prioritizes distant others. But when loyalty is construed more broadly, such as through shared identity or social ideals (as reflected in group loyalty), it may actually reinforce support for equitable and impact-driven altruism. These findings challenge traditional assumptions that loyalty is necessarily at odds with moral expansiveness and equitable altruism (3), and highlight the importance of how moral group boundaries are drawn. Figure 4 presents a visual depiction of the associations between group loyalty on the MAC-Q with scores on the EAIS-EX across the three samples.

## Discussion

Building on philosophical and theoretical perspectives that emphasize both the parochial roots of human morality (1, 2, 6, 62, 66, 67) and its capacity for expansion (13, 14, 18, 21, 24, 25, 64), the present research examined the moral beliefs and values that support and constrain both exceptional and everyday forms of prosociality. In addition to focusing on prosociality when broadly construed as the amount of money people report donating to charity or the amount of time they report volunteering, we focused in particular on equitable altruism (helping others regardless of distance or similarity), effective altruism (maximizing welfare through high-impact giving), and forms of altruism that combine both equity and effectiveness, a combination widely viewed as critical for addressing many of the world's most urgent challenges (17, 39, 41, 42, 44). Furthermore, while prior research has shown that most adults tend to favor helping those who are socially or geographically close (12, 26–28)—prioritizing proximity and familiarity over need or consequence—we investigated the moral values that underlie altruism in two rare populations who defy these tendencies: EAs and Extraordinary Altruists (XAs), alongside a demographically similar control group.

While EAs and XAs both incur costs to help strangers for whom typical adults feel little concern (44), they are often seen as embodying distinct prosocial orientations: EAs as strategic, utilitarian, and outcome-focused (17, 39, 41); XAs (operationalized here as nondirected, living organ donors) as sacrificial and person-centered (34, 37). And, although prior research has begun to map the moral values of XAs (22, 23), this study is the first to empirically examine the moral architecture of self-identified EAs. Our findings largely support that both groups are morally expansive, while also revealing important differences in their underlying values that align with the subtle nuances of their prosocial orientations. As such, studying these populations together reveals distinctions in their moral beliefs and values that contributes to our growing understanding of the outer boundaries of human prosociality (22, 23, 41), demonstrating that multiple moral pathways may support engagement in altruism that not only transcends social boundaries, but also prioritizes effectiveness. And, although EAs and XAs are rare, they are not inaccessible. Unlike moral heroes like prominent civil rights leaders (69), they are ordinary individuals whose values offer practical insight into how equitable and effective altruism might be more widely cultivated through intervention.

Moreover, the present findings challenge prevailing theoretical accounts of loyalty as inherently parochial and in tension with impartial prosociality (3, 19, 21, 45). While past work has emphasized that strong commitments to close others often hinder concern for distant beneficiaries, our results suggest a more nuanced picture which we discuss in greater detail below. Taken together, the findings illuminate the psychological foundations of real-world exceptional altruism and offer insight into the diverse moral commitments that can motivate more impartial (expansive) and impactful (effective) prosocial action in an increasingly interconnected—and morally demanding (40)—world.

### Moral expansiveness and IB are key to understanding both equitable and effective altruism

Across analyses, two moral orientations—moral expansiveness and IB—emerged as central to understanding impact-maximizing altruism that reaches beyond conventional social boundaries. These values were not only elevated in both altruistic groups compared to controls but also predicted stronger support for equitable and effective altruistic attitudes, judgments, and, in some cases, behaviors. The consistent role of moral expansiveness supports theories that emphasize a broadened moral circle as a core psychological mechanism for impartial altruism (21–23, 25). Notably, participants who assigned moral worth to a broader range of entities, including stigmatized groups, outgroup members, and non-human animals, were more likely to endorse helping behaviors untethered by familiarity, identity, or proximity. These findings lend empirical support to philosophical accounts that see moral progress as an expansion of concern (e.g. Singer's “expanding circle” framework (14, 18)) while also reinforcing the idea that, although morality may have evolved to promote ingroup cooperation (3, 62), moral rights—when extended more broadly—can support prosociality for the benefit of strangers, distant others, and global welfare.

Importantly, our findings also *build upon* existing theories that center moral expansiveness (24) by showing that extending concern more broadly does not only predict more equitable altruism, but also more effective altruism. That is, those who endorsed more expansive moral circles were not only more likely to help others regardless of proximity, but also more likely to prioritize actions that produce the greatest benefit. This may reflect the fact that those most in need of help are often distant—geographically, socially, or psychologically (70). As a result, when individuals extend moral concern beyond conventional boundaries, concern for high-impact, welfare-maximizing altruism may naturally follow. For instance, when deciding whether to help one compatriot or two individuals in a distant country, those higher in moral expansiveness may see the choice as a more straightforward tradeoff (e.g. two lives versus one) rather than a conflict between aiding someone they feel stronger concern for versus someone more psychologically remote.

Notably, prior research has found that while the real-world altruism of organ donors aligns more closely with equity than with explicit concern for effectiveness, XAs still show greater prioritization of effective outcomes in their behavior (43). It was speculated that this might reflect a more implicit orientation toward welfare-maximization, perhaps as a downstream consequence of their expansive moral concern. As mentioned above, our findings here support this interpretation: a more expansive moral circle—though not explicitly focused on impact—may nonetheless promote effective altruism indirectly, by increasing the moral salience of those most in need, who are often the most distant or overlooked (40).

At the same time, impartial beneficence (i.e. a principled commitment to maximizing welfare irrespective of who benefits) also emerged as a particularly strong predictor of effective altruism. Among controls, it outperformed moral expansiveness in predicting support for impact-driven altruistic outcomes. While moral expansiveness reflects the breadth and depth of concern, impartial beneficence reflects the prioritization of that concern: a moral commitment to helping those who stand to benefit most, even when doing so requires trading off aid to those who are close (32). This distinction may be especially important in understanding the moral drivers of outcome-oriented forms of altruism. Although both EAs and XAs endorsed IB more strongly than controls, this value was particularly pronounced among EAs, highlighting a key difference in their moral orientation. Whereas XAs extend concern broadly in ways that more explicitly support equitable helping, EAs appear to couple this concern with an explicit and remarkably strong moral commitment to effectiveness—setting them apart not only from the general population but also from other altruists whose motivations may be more rooted in compassion than calculus (43).

### Might values that bind support rather than inhibit boundless altruism?

Beyond examining orientations toward moral impartiality, we also examined finer-grained distinctions in specific moral values. While, in line with our findings, the “individualizing” moral value of harm reduction generally associated positively with equitable and effective altruism across populations, our findings challenge dominant accounts of loyalty as inherently parochial and antithetical to impartiality (3, 20, 21). While familial loyalty was consistently associated with reduced support for equitable and effective altruism, suggesting that strong kin commitments may compete with expansive moral concern, group loyalty told a different story. Across all three samples, including the general population, group loyalty often predicted greater support for both altruistic equity and effectiveness. This pattern underscores a distinction between narrow, kin-based loyalty, and loyalty to more abstract or inclusive groups.

These findings speak directly to ongoing debates surrounding theories of moral pluralism (e.g. Moral Foundations Theory (19), Morality-as-Cooperation Theory (3)) about the role of binding values like loyalty in supporting or inhibiting equitable prosociality. They also align with recent work showing that moral concern for close others correlates positively with moral concern for those who are distant (65, 71), as well as with emerging insights suggesting that even the most extraordinary altruists nonetheless maintain fulfilling close relationships (63). Rather than treating loyalty as uniformly restrictive, our results contribute to this growing narrative and suggest that loyalty's influence may depend on the breadth and content of the group to which one feels loyal and whether commitments are made merely to one's kin or to one's broader community (48).

We offered one possible explanation for this pattern above: for altruistic individuals in particular, group loyalty may reflect identification with expansive moral communities—such as “humanity,” “those in need,” or “sentient beings”—that serve to expand, rather than contract, the moral circle. Indeed, we find that altruists extend greater moral concern to distant entities than controls. Furthermore, an exploratory analysis suggested that the effect of group loyalty on expansive altruism was descriptively stronger among extraordinary altruists with more expansive moral circles, though this interaction did not reach significance, potentially due to limited statistical power.^b^ However, the fact that group loyalty related positively with equitable and effective altruism even among controls suggests this pattern may reflect a more fundamental feature of human moral psychology that challenges our current understanding.

One possibility is that the very values that support strong bonds within communities, such as commitment to shared norms, signaling trustworthy intentions, and engaging in coordinated cooperative efforts (2, 5, 72), may also lay the groundwork for more expansive altruistic behavior. That is, the social and cognitive capacities that underlie group loyalty, including the ability to track reputations, adhere to collective goals, and uphold communal traditions, may not only be instrumental in maintaining cohesive alliances but also reinforce prosocial motivations that extend beyond one's immediate circle. Indeed, existing theoretical accounts support that engaging in acts that signal loyalty and integrity within a group can bolster trust and status, both of which are adaptive for navigating complex social systems (2, 73, 74). Yet, when group identity is not narrowly defined (e.g. extending beyond familial obligations to one's group or even farther), these same mechanisms may support commitments to more expansive moral obligations, amplifying concern for distant others and reinforcing a desire to contribute meaningfully to broader societal good. Aligned with this possibility, despite the fact that helping distant others can be viewed with suspicion in zero-sum contexts (29, 31, 32), altruists who express concern beyond their immediate circles, particularly when no direct tradeoff is involved, are often seen as more trustworthy and morally admirable (30, 75, 76). Similarly, cross-cultural evidence shows that national identity during the COVID-19 pandemic, a proxy for group loyalty, predicted adherence to prosocial public health behaviors (77). As such, the traits that bind communities together may also help bridge them outward, suggesting that loyalty and expansive altruism can be mutually reinforcing rather than mutually exclusive.

Building on the present findings, future research should investigate why and under what conditions a value often considered parochial—group-level loyalty—predicts equitable, impact-oriented altruism. One promising direction is to examine how the *content* and *scope* of group identity shape this relationship. Experimental studies could manipulate whether individuals identify with narrowly defined collectives (e.g. family, neighborhood) versus broader, more abstract moral communities (e.g. humanity, all sentient beings) and assess whether loyalty to these groups facilitates more equitable helping. In tandem, further work could explore the mechanisms at play—such as whether expansive altruism emerges from internalized moral commitments to inclusive groups or from reputational strategies that signal trustworthiness, moral integrity, and alignment with cooperative norms. Such work would shed light on when group loyalty becomes a bridge rather than a boundary, helping clarify how values rooted in communal cohesion might also foster expansive moral concern and effective prosocial action.

### Do equitable and effective altruism rely on different moral underpinnings?

A central contribution of this work is its ability to disentangle whether separate moral architectures support equitable versus effective altruism. Although both EAs and extraordinary altruists (XAs) engage in impartial forms of giving, extending help to strangers regardless of proximity or identity, they appear to arrive at this commitment through overlapping albeit distinct moral routes. EAs displayed a uniquely high endorsement of IB and were more accepting of instrumental harm (IH), aligning with a utilitarian outlook that prioritizes maximizing good outcomes (38, 41). Some caution is warranted in interpreting these findings. Our outcome measures focused primarily on attitudes and behaviors aligned with IB, not instrumental harm, so we cannot directly assess whether IH endorsement translates into IH-aligned behavior. Moreover, because EA is rooted in consequentialist philosophy, high scores on IH among some EAs may reflect signaling of a broader consequentialist identity rather than an intention to act on such beliefs. Nonetheless, the results are noteworthy, as they suggest that EAs on balance may endorse consequentialist principles in ways that go beyond the movement's formal ethical commitments (78).

EAs also deprioritized traditional cooperative values such as reciprocity and property rights, suggesting a moral style that is more willing to revise or override social norms in service of impact (2). In contrast, XAs expressed similarly expansive moral concern but placed greater value on conventional cooperative norms, including reciprocity and group loyalty. Their altruism appears less rooted in abstract moral calculation and more closely aligned with compassionate responsiveness and relational moral sensibilities. This pattern suggests that impartial altruism need not arise from a rejection of typical moral commitments. Instead, and as discussed in detail above, it may be scaffolded by them, especially when those commitments are extended beyond narrow ingroups to encompass a broader moral community.

Importantly, the contrast between XAs and EAs does not imply that effective altruism *requires* rejecting core moral values, even those associated with ingroup cooperation. EAs downplay norms like reciprocity, property rights, and authority, and endorse instrumental harm more than other groups, yet still uphold core values like harm reduction and group loyalty at levels comparable to the general population. This supports that commonplace morality and demanding consequentialist reasoning can coexist within a single moral framework. XAs, defined by their profoundly equitable act of donating a kidney to a stranger, also prioritize more effective causes than controls (though to a lesser extent than EAs; see related findings (43)) and show stronger endorsement of IB. Notably, they do so without endorsing instrumental harm and while maintaining many core moral values typical of the general population. In contrast, EAs were the only group to significantly endorse instrumental harm, raising questions about whether some consequentialist commitments extend beyond the EA movement's stated values (39). Yet, both populations challenge the idea that equitability or effectiveness must conflict with everyday moral norms, and reveal that multiple moral and psychological architectures, some more consequentialist (among EAs) and others more relational (among XAs), can support both.

### Limitations

Despite the strengths of the present investigation, the findings should be interpreted in light of several limitations. First, although this study provides the first systematic comparison of self-identifying EAs, extraordinary altruists, and demographically similar controls across a range of moral values and prosocial outcomes, all analyses are correlational. As such, *causal* inferences about the roles of specific moral beliefs in shaping altruistic behavior cannot be drawn. Second, despite rigorous pre-registration and recruitment, the rarity of the altruistic populations resulted in modest sample sizes (particularly for the XAs, of whom there were 65), limiting statistical power of regression models within this sample. While we took steps to minimize both false positives (e.g. Bonferroni corrections) and false negatives (e.g. emphasizing standardized slopes and CIs over *P*-values for “trending” effects), we advise caution when interpreting nonsignificant results. Further, while the measures of moral values are well-validated and while their predictive patterns were largely consistent across samples here, larger altruistic samples would also enable formal tests of measurement invariance to ensure that altruists and nonaltruists interpret morally loaded measures similarly. Third, because EAs are defined by adherence to a philosophical movement and XAs by having performed a high-stakes altruistic act, it is possible that EAs exhibit greater ideological coherence than XAs, who may be more heterogeneous in their motivations and worldview. While we do not find evidence of heterogeneity of variance for religiosity (Levene’s *F*(2,357) = 1.88, *P* = 0.154) nor political ideology (Levene’s *F*(2,357) = 1.85, *P* = 0.158) across groups, we acknowledge that other unmeasured characteristics may vary more widely among XAs than among EAs.

Another potential limitation is that some predictors (e.g. IB, Moral Expansiveness) and outcomes (e.g. EA Interest Scale, BDT) occupy overlapping conceptual space. Though they differ in abstraction (principle versus intention), framing (abstract concern versus real sacrifice), and modality (self-report versus monetary decisions), some associations may reflect conceptual or methodological proximity. However, all correlations were moderate (none >0.52), indicating substantial unique variance. Moreover, the Donation Task involves real tradeoffs, and the EAIS distinguishes impartiality from cost-effectiveness, reducing risk of tautology. Nonetheless, some caution in interpreting predictive strength remains warranted.

Although some EAs (and controls) were based in Africa, Asia, and South America, most participants came from WEIRD (79) populations. This reflects the demographics of the altruistic populations studied (41, 80) but limits generalizability, as values like fairness, impartiality, and loyalty vary across cultures (52, 60). Structural factors such as socioeconomic status (SES) and education also shape the expression and feasibility of altruistic behavior. Lower-SES individuals show somewhat reduced interpersonal prosociality on average (81, 82), in part because limited resources can constrain impartial or high-impact altruism (83). Future research should test whether these patterns generalize to more socioeconomically and culturally diverse populations and examine how structural conditions shape opportunities for equitable and effective altruism.

A related limitation is the gender imbalance across groups: EAs were mostly male, XAs mostly female. These reflect real-world trends. More men identify as EAs, and more women become nondirected organ donors (35, 39). Although our control group was demographically similar, EA–XA comparisons may still partly reflect gender-linked variation. Since gender tracks group membership in the real world, controlling for it could obscure key differences. Still, EAs and XAs often showed similar outcomes, and both are rare in the general population, suggesting shared psychological or motivational factors likely underlie their distinct altruistic profiles.

Finally, our findings raise important questions for future work on the measurement of core moral values. For example, across several analyses, fairness, when measured via standard tools like the MFQ and MAC-Q, was inconsistently predictive of altruistic outcomes and even unexpectedly low among altruists. This may reflect measurement limitations rather than theoretical failure. As others have demonstrated (e.g. Atari et al. (60); Zakharin and Bates (84)), existing scales often conflate fairness-as-equality with fairness-as-merit or proportionality. If effective and extraordinary altruists emphasize need-based equity over performance-based proportionality, their fairness intuitions may not be captured by standard instruments. Importantly, both altruistic samples strongly endorsed IB, which reflects a deep concern for moral equality and fairness in its broadest sense (38). These results suggest that altruists *do* value fairness, but that current measurement tools may fail to capture the form of fairness most relevant to their moral outlook. More nuanced instruments are needed to disentangle these dimensions and clarify how fairness supports, or fails to support, different forms of altruism. More broadly, the generally lower endorsement of many moral values among EAs raises the possibility that widely used moral measures may not adequately capture the psychological profiles that support the most exceptional forms altruism.

## Conclusion

In sum, this work sheds light on the moral architecture that underpins expansive and impactful forms of altruism. By examining populations who engage in high-cost and high-impact giving, we reveal how specific value systems, such as broad moral concern and IB, are associated with attitudes, judgments, and real-world behaviors that reach beyond conventional boundaries of proximity, familiarity, and deservingness. The findings challenge the idea that impartiality necessarily comes at the expense of group loyalty, showing instead that expansive moral concern can coexist with, and perhaps even be supported by, broader forms of commitment to those who are close. Moreover, they challenge the notion that high-impact giving necessitates abandoning cooperative norms or embracing even the dark side of utilitarianism (i.e. instrumental harm), as high-impact prosocial behavior can be arrived upon through moral expansiveness and principled impartiality alone. As the scale of global suffering calls for increasingly equitable and effective action, understanding the values that motivate such efforts offers a step toward cultivating a more compassionate and strategically engaged moral community.

## Materials and methods

### Open science practices

This article examines moral beliefs and values as well as prosociality among EAs, Extraordinary Altruists, and general population controls. It is part of a larger project leveraging the same extensive dataset to address a variety of distinct research questions that will be presented in separate articles. Due to the uniqueness and rarity of the subject groups, data for all related projects were collected simultaneously to ensure efficiency and make the best use of our participants’ time. Additional articles from this dataset will explore topics such as empathy and reasoning, creative and imaginative capacity, close relationship quality, future-oriented thinking, social attitudes, and attitudes toward animals and nature within the same samples. Further details about the full dataset and the broader project are available in a “Read Me” file on the Open Science Framework (OSF) at https://osf.io/pyjnk/?view_only=151c6bdcfad34c6ebea9afc934063b64. The OSF also houses additional relevant materials, including the raw dataset (featuring all measured variables across the related projects), the cleaned dataset containing the variables specific to this article, the full surveys administered to each sample, and analysis scripts related to the current article.

Sample characteristics, as well as the hypotheses and analyses for both within-group and between-group comparisons, were pre-registered on aspredicted.org. Pre-registrations for within-group analyses (e.g. relationships between moral beliefs/values and prosociality) are available for the EA sample (https://aspredicted.org/vkb5-ngxv.pdf), the XA sample (https://aspredicted.org/nd3q-ysxm.pdf), and the control sample (https://aspredicted.org/q4qr-8vms.pdf). We pre-registered both correlations and regressions but prioritized the regressions in the main text, as they offer a more stringent, confirmatory test by accounting for shared variance among predictors. Pre-registrations for between-group analyses (e.g. comparisons across EA, XA, and control groups on measures of moral beliefs/values) can be found at https://aspredicted.org/xfsx-7dmd.pdf. We pre-registered one-way ANOVAs with Bonferroni post hoc tests, which are reported here. Although we allowed for the option of less conservative corrections in the pre-registration, we retained Bonferroni to reduce false positives and report effect sizes and 95% CIs for transparency. Across all four pre-registrations listed above, we pre-registered several exploratory analyses unrelated to the current research questions (e.g. clustering, NLP), which are being pursued separately as part of a broader project. Additional recruitment criteria specific to the demographic matching of controls to their target groups were pre-registered at https://aspredicted.org/czjn-yb3p.pdf. Since EA recruitment relied on social media, Slack channels, and forums, further exclusion criteria to identify and exclude fraudulent responses (i.e. survey bots) were pre-registered at https://aspredicted.org/vjnk-yhgb.pdf. Data from responses excluded based on these criteria are publicly accessible on the OSF.

### Participants

An a priori power analysis determined that a sample of *N* = 319 subjects per participant group would provide 95% power to detect an effect size of *r* = 0.2 (two-tailed test, *α* = 0.05). Given the specialized nature of the two special population samples, we planned to collect as many cases as possible within 90 days of active data collection or until *N* = 319 was reached, whichever came first.

#### Sample 1: EAs

We recruited EAs using social media channels associated with the EA movement (e.g. The “Giving What We Can” Slack Channel, The Effective Altruism Forum, the Twitter [X] account of Peter Singer, a prominent figure within the EA movement). Participants who self-identified as EAs were directed to complete a survey on Qualtrics, receiving $15 for their participation. After accounting for exclusions made on the basis of our pre-registered criteria for fraudulent responding (e.g. nonsensical or duplicate open-ended responses), 206 self-identifying EAs completed the survey during the 90 days of active recruitment. Of the 206 respondents, 87 were excluded for failing attention checks or possessing duplicate IP addresses, leaving *N* = 119 EAs in sample 1. Sensitivity analysis indicated 95% power to detect *r* = 0.32 or 80% power to detect *r* = 0.25 (two-tailed test, *α* = 0.05).

#### Sample 2: extraordinary altruists (XAs)

Extraordinary altruists (i.e. XAs-nondirected kidney, liver, and tissue donors) were recruited through an existing network of XAs maintained by a research laboratory at a prominent university in the Eastern United States. Participants who had donated an organ to a stranger, and whose donations were independently verified, completed the survey on Qualtrics and received $15. Of the 66 respondents who completed the survey, only one was excluded for failing attention checks, resulting in *N* = 65 donors (57 kidney donors, 7 kidney/liver double-donors, and 1 kidney/marrow double-donor). This sample achieved 95% power to detect *r* = 0.43 or 80% power to detect *r* = 0.34 (two-tailed test, *α* = 0.05). Though a small sample, this sample size aligns with similar research on nondirected living donors and reflects the rarity of such costly altruism (22, 36, 37).

#### Sample 3: general population controls

The control sample consisted of English-speaking general population participants who (i) did not identify as EAs and (ii) had not donated an organ or body tissue to a stranger. The target sample size was designed to match the combined size of the two special population samples (samples 1 and 2). We recruited control participants via Prolific in two phases, matching demographics to sample 1 (EAs) and sample 2 (XAs) respectively. In phase 1, we aimed to recruit *N* = 119 controls matched to sample 1 on nationality, gender, and age. To account for exclusions, *N* = 139 participants were recruited. One additional participant completed the survey without submitting for remuneration and was retained in the raw dataset. After excluding 21 participants who failed more than three of the 13 attention checks, data from *N* = 109 participants were retained.

In phase 2, we aimed to recruit *N* = 65 controls matched to sample 2 on nationality, gender, age, and race/ethnicity. For the phase 2 controls, Prolific's quota sampling functionality enabled us to include the additional dimension of race/ethnicity in the matching criteria due to the smaller number of nationalities represented in the XA sample. To allow for exclusions, *N* = 75 participants were recruited. Again, one additional participant completed the survey without submitting for remuneration and was retained in the raw dataset. Nine participants were excluded for failing attention checks, leaving *N* = 67 participants in this phase. Across both phases, no participants had duplicate IP addresses. In total, the control sample comprised *N* = 176 participants, achieving 95% power to detect *r* = 0.27 or 80% power to detect *r* = 0.21 (two-tailed test, *α* = 0.05).

#### Combined sample

Across all samples, data were collected from *N* = 360 participants (*N* = 119 EAs, *N* = 65 donors, *N* = 176 controls), achieving 95% power to detect *r* = 0.19 or 80% power to detect *r* = 0.15 (two-tailed test, *α* = 0.05). Table S1 provides demographic details for each sample.

### Materials and procedure

This research includes data from human participants and the procedures were approved by the University at Albany, SUNY IRB (protocol number: 22X187). After providing informed consent, participants completed a Qualtrics survey containing well-validated measures assessing the predictors (i.e. measures of moral beliefs and values) and outcomes (i.e. equitable and effective prosociality) in a randomized order. Demographic data and debriefing followed. Payment details were collected separately to protect anonymity, with samples 1 and 2 receiving $15 gift cards via email and sample 3 paid directly through Prolific. Table S2 comprehensively covers key information for each of these measures in detail, including example items, reliability statistics, scoring procedures, and scale interpretation. Additional information on the key outcomes capturing moral beliefs and values is located in the Supplementary Materials. The full text of these items can be located with the full surveys on the OSF page.

## Supplementary Material

pgaf326_Supplementary_Data

## Data Availability

Details about the full dataset and the broader project are available in a “Read Me” file on the OSF at https://osf.io/pyjnk/?view_only=151c6bdcfad34c6ebea9afc934063b64. The OSF also houses additional relevant materials, including the raw dataset (featuring all measured variables across the related projects), the cleaned dataset containing the variables specific to this article, the full surveys administered to each sample, and analysis scripts related to the current article.

## Notes

a While both MFT and MAC are pluralistic frameworks in moral psychology, each has received critiques: MAC for its theoretical scope (e.g. see Bloom's commentary on Curry's 2019 article in *Current Anthropology* (2)), and MFT for its conceptual and measurement challenges (e.g. see Gray and colleagues’ Theory of Dyadic Morality (47)). We draw upon both MFQ and MAC as examples of moral pluralism without taking a stance in these debates.

b For exploratory purposes, we tested whether overall moral concern, as measured by the Moral Expansiveness Scale (MES), moderated the association between group-based loyalty and support for equitable altruism (EAIS-EX). This outcome was selected given that group-based loyalty significantly and positively predicted equitable altruism across all three samples. We observed a pattern consistent with moderation among XAs, such that the relationship between group loyalty and equitable altruism was stronger for XAs with more expansive moral concern. However, this interaction did not reach statistical significance, likely due in part to limited power in the XA sample. No evidence for moderation emerged among EAs or controls (see Table S7 and Fig. S1 for full results). These findings suggest a possible role for expansive moral concern in enabling parochial values like loyalty to support equitable altruism—particularly among individuals already committed to altruistic ideals.

## Preprints

This manuscript was posted on a preprint: https://osf.io/preprints/psyarxiv/pafuw_v1?view_only=.
