# Supplementary material for: Mapping the moral architecture of effective and extraordinary altruism
Source: PNAS Nexus. 2025 Oct 22;4(10):pgaf326. doi: 10.1093/pnasnexus/pgaf326 (PMC12560819; doi:10.1093/pnasnexus/pgaf326)
Supplement: pgaf326_Supplementary_Data [file pgaf326_supplementary_data.pdf]

Mapping the Moral Architecture of Effective and Extraordinary Altruism

Supplementary Materials

Table of Contents

Demographic Information for All Samples.. page 3

Key Detail on Measures.. page 4

Sample Differences in Moral Beliefs and Values..page 6

Sample Differences in Finer-Grained Moral-Circle Distinctions...page 8

Regressions Between Measures of Morality and Prosociality..page 9

Bivariate Correlations Between Measures of Morality and Prosociality.................................... page 11

Additional Detail on Measures of Moral Beliefs and Values..page 12

Moderation Analyses...page 14

Supplementary References..page 15

Survey Questions...page 16

Demographic Information for All Samples

Table S1. Information about the three samples

Parameter		Sample 1: Effective Altruists (EAs)	Sample 2: Extraordinary Altruists (XAs)	Sample 3: Controls
Information About the Populations	Population Description	Individuals who self-identify with the effective altruism philosophical movement	Non-directed living organ donors	Ordinary adults
	Population Characteristics	Aligning closely with both equity and effectiveness, EAs seek to maximize the impact of their altruistic actions through charitable donations that help strangers beyond their immediate community	XAs have voluntarily donated an organ—while living—to a complete stranger they have never met; while the defining characteristic of this population aligns most-closely with equity, XAs tend to behave more effectively than controls on prosocial laboratory tasks (see below)	Controls were recruited using quota sampling to be demographically similar to Samples 1 and 2
	Scale of Altruism	EAs, on average, reported donating 15.6% of their yearly income to charitable causes	XAs comprised 57 kidney, 7 kidney/liver, and 1 kidney/bone marrow donor(s)	Controls, on average, reported donating only 3.7% of their yearly income to charitable causes
	Scoring Patterns on Laboratory Metrics of Equitable and Effective Prosociality (see Law et al., 2025[43])	EAs have been shown to score higher than controls on measures of both equitable and effective prosociality, and higher than XAs and controls on measures of effective prosociality	XAs have been shown to score higher than controls on measures of equitable prosociality, and on behavioral—though not attitudinal—measures of effective prosociality	Controls score lower than both EAs and XAs on measures of equitable prosociality; they also score lower than EAs on both attitudinal and behavioral metrics, and lower than XAs on behavioral metrics, of effective prosociality.
	Recruitment Channels	EA slack channels, forums, and social media groups	A validated research database of living organ donors	Prolific
	Criteria for Inclusion in Sample Category	Self-identifying as an effective altruist, and, by nature of where recruitment was carried out, participation in EA-affiliated online spaces	Having donated an organ to a stranger while living	Being an English-speaking Prolific worker who meets the demographic matching criteria laid out in the Methods
Gender	N_{Total}	119	65	176
	N_{Male}	79	22	97
	N_{Female}	36	42	77
	N_{Other}	4	1	2
Race	N_{White}	73	59	117
	N_{Black}	17	0	22
	N_{Asian}	13	3	19
	$N_{Multiracial}$	8	2	11
	$N_{OtherRace}$	8	1	7
Geographic Region	$N_{UnitedStates}$	46	61	103
	N_{Canada}	2	4	8
	$N_{UnitedKingdom}$	13	0	17
	$N_{AfricanContinent}$	21	0	13
	$N_{AsianContinent}$	8	0	8
	$N_{EuropeanContinent}$	21	0	19
	$N_{SAmericanContinent}$	1	0	1
	$N_{Australia\&NZ}$	7	0	7
Age (years)	M_{age}	31.9	53.0	37.2
	SD_{age}	8.8	12.5	12.1
Income[1]	M_{income}	3.0	4.5	3.0
	SD_{income}	1.9	1.5	1.6
Education[2]	$M_{education}$	7.9	8.0	6.9
	$SD_{education}$	1.9	1.8	2.0

Note. [1]Income was captured on a 1 "less than $25,000" – 6 "$150,000 or more" scale. On average, EAs and Controls fell into the "$50,000 – $74,999" bracket, while XAs fell into the "$75,000 – $100,000" bracket. [2]Education was captured on a 1 "no schooling completed" – 11 "doctorate degree" scale. On average, EAs and XAs obtained a bachelor's degree or equivalent, while controls obtained an associate's degree or equivalent.

Key Detail on Measures

Table S2. Key information on the measures capturing moral beliefs and values as well as equitable and effective prosociality

Measure, Example Item, Reliability	High Score Interpretation, Scale Points	Scale Points
**Moral Beliefs and Values (Predictor)**		
**Moral Foundations Questionnaire (MFQ[20])**<br>†*MFQ Harm Reliability:* α=0.60<br>†*MFQ Fairness Reliability*: α=0.68<br>††*MFQ Loyalty Reliability*: α=0.77<br>**MFQ Authority Reliability*: α=0.82<br>**MFQ Purity Reliability*: α=0.85	Greater moral prioritization of the prosocial values of harm and fairness, the parochial value of loyalty, and, for exploratory purposes, the values of authority and purity	0-5 (Likert)
**Morality as Cooperation Questionnaire (MAC-Q[2])**<br>†*MAC-Q Fairness Reliability*: α=0.73<br>††*MAC-Q Familial Loyalty Reliability*: α=0.91<br>††*MAC-Q Group Loyalty Reliability*: α=0.86<br>**MAC-Q Reciprocity Reliability*: α=0.81<br>**MAC-Q Heroism Reliability*: α=0.86<br>**MAC-Q Deference Reliability*: α=0.91<br>**MAC-Q Property Reliability*: α=0.66	Greater moral prioritization of the prosocial value of fairness, the parochial values of familial loyalty and group loyalty, and, for exploratory purposes, the values of reciprocity, heroism, deference to authority, and property rights	0-100 (Slider)
**Oxford Utilitarianism Scale (OUS[60])**<br>†*OUS Impartial Beneficence Reliability*: α=0.77<br>**OUS Instrumental Harm Reliability*: α=0.80	Greater moral endorsement of impartial beneficence (IB) and instrumental harm (IH), the sub features of utilitarianism—pre-registered hypotheses pertain to IB, while those pertaining to IH are exploratory	1-7 (Likert)
**Moral Expansiveness Scale (MES[25])**<br>†*MES Overall Moral Concern:* α=0.95<br>*MES Family and Friends:* α=0.68<br>*MES Ingroups:* α=0.80<br>*MES Revered Humans:* α=0.83<br>*MES Stigmatized Humans:* α=0.75<br>*MES Outgroups:* α=0.85<br>*MES High-Sentience Animals:* α=0.90<br>*MES Low-Sentience Animals:* α=0.89<br>*MES Plants:* α=0.93<br>*MES Environments:* α=0.88<br>*MES Villains:* α=0.94	Greater moral concern for all entities taken together (i.e., "Overall"), and separately for family/friends, ingroups, revered humans, stigmatized humans, outgroups, high-sentience animals, low-sentience animals, plants, environments, and villains	0= Outside the Moral Boundary, 1 = Fringes of Moral Concern, 2 = Outer Circle of Moral Concern, 3 = Inner Circle of Moral Concern
**Measures of Equitable and Effective Prosociality (Outcome)**		
**Measure, Example Item, Reliability**	**High Score Interpretation, Scale Points**	
**Moral Judgment Vignettes (MJV[31])**<br>8 short vignettes (e.g., "To what extent was it morally acceptable for the person in the story to donate money to the UN charity for people in another country instead of 1 friend?"; α=0.93	Greater moral acceptability of welfare-maximizing altruism directed towards distant beneficiaries (**equitable and effective altruism captured**)	1-9 (Likert)
**Social Discounting Task (SDT[88])**<br>27 hypothetical decisions ("Would you prefer $80 for yourself or $85 dollars for [a mutual friend or acquaintance]")	Greater preference for larger rewards for distant others over smaller rewards for oneself (**equitable and effective altruism captured**)	Scored 0-27 (The number of choices favoring a target other than oneself)
**Behavioral Donation Task (BDT**)**<br>16 consequential choices between lifesaving (e.g., The Malaria Consortium) and life-improving (e.g., National Caregiving Foundation) charitable causes	Greater number of donations to effective vs. ineffective causes (**effective altruism captured**)	Scored 0-16 (The number of choices to donate actual resources [USD] to an effective versus ineffective cause)
**Effective Altruism Interest Scale (EAIS[41])**<br>*Expansive Altruism (EAIS-EX):* 6 items (e.g., "I am willing to make significant sacrifices for people in need that I don't know and will never meet."); α=0.87	Greater endorsement of **effective** (effectiveness focus subscale) and **equitable** (expansive altruism subscale) altruism	1-7 (Likert)

Effectiveness Focus (EAIS-EF): 6 items (e.g., "It would be the right choice to refrain from helping one person if that makes it possible to help a larger number of people."); *α*=0.82		
**Reported Real-World Charitable Action (RWCA**)**<br>*RWCA Money:* "In a given year, what percentage (out of 100) of your yearly income do you donate to charity?"<br>*RWCA Time:* "In a given year, what percentage (out of 100) of your time do you devote towards volunteering to help others?"	Greater proportions of income devoted to philanthropic causes (for income) and volunteerism (for time) in a given year (**general prosociality measured**)	0%-100% (Slider)

Note. †Predictor hypothesized to relate positively to measures of equitable and effective altruism. ††Predictor hypothesized to relate negatively to measures of equitable and effective altruism. *Variable measured for exploratory purposes. **Measure developed for the current research.

Sample Differences in Moral Beliefs and Values

Table S3. Results from one-way ANOVAs with Bonferroni-corrected post-hoc tests comparing Samples 1-3 on measures capturing moral beliefs and values

Sample	Prosocial Moral Beliefs and Values
**Moral Valuation of Care and Harm Reduction (MFQ–Harm)**	
Omnibus	$F(2, 357) = 2.57$, $p = .078$, $\eta^2p = .014$, 95%CI [0.00, 0.04]
EA vs. Control	$t(357) = -2.25$, $p = .075$, Cohen's $d = -0.267$, 95%CI [-0.50, -0.03]
XA vs. Control	$t(357) = -0.50$, $p > .999$, Cohen's $d = -0.073$, 95%CI [-0.36, 0.21]
EA vs. XA	$t(357) = -1.26$, $p = .624$, Cohen's $d = -0.195$, 95%CI [-0.50, 0.11]
**Moral Valuation of Fairness (MFQ–Fairness)**	
Omnibus	$F(2, 357) = 8.52$, $p < .001$, $\eta^2p = .046$, 95%CI [0.01, 0.09]
EA vs. Control	$t(357) = -3.44$, $p = .002$, Cohen's $d = -0.408$, 95%CI [-0.64, -0.17]
XA vs. Control	$t(357) = -3.29$, $p = .003$, Cohen's $d = -0.478$, 95%CI [-0.77, -0.19]
EA vs. XA	$t(357) = 0.45$, $p > .999$, Cohen's $d = 0.070$, 95%CI [-0.23, 0.37]
**Moral Valuation of Fairness (MAC-Q–Fairness)**	
Omnibus	$F(2, 357) = 3.85$, $p = .022$, $\eta^2p = .021$, 95%CI [0.00, 0.06]
EA vs. Control	$t(357) = -2.52$, $p = .036$, Cohen's $d = -0.300$, 95%CI [-0.53, -0.07]
XA vs. Control	$t(357) = -1.92$, $p = .167$, Cohen's $d = -0.279$, 95%CI [-0.57, 0.01]
EA vs. XA	$t(357) = -0.13$, $p > .999$, Cohen's $d = -0.021$, 95%CI [-0.32, 0.28]
**Overall Moral Concern (MES)**	
Omnibus	$F(2, 357) = 30.90$, $p < .001$, $\eta^2p = .147$, 95%CI [0.08, 0.21]
EA vs. Control	$t(357) = 7.36$, $p < .001$, Cohen's $d = 0.874$, 95%CI [0.63, 1.12]
XA vs. Control	$t(357) = 5.02$, $p < .001$, Cohen's $d = 0.728$, 95%CI [0.43, 1.02]
EA vs. XA	$t(357) = 0.94$, $p > .999$, Cohen's $d = 0.146$, 95%CI [-0.16, 0.45]
**Moral Judgments of Impartial Beneficence (OUS–IB)**	
Omnibus	$F(2, 357) = 37.90$, $p < .001$, $\eta^2p = .175$, 95%CI [0.11, 0.24]
EA vs. Control	$t(357) = 8.70$, $p < .001$, Cohen's $d = 1.032$, 95%CI [0.79, 1.28]
XA vs. Control	$t(357) = 2.54$, $p = .035$, Cohen's $d = 0.368$, 95%CI [0.08, 0.66]
EA vs. XA	$t(357) = 4.30$, $p < .001$, Cohen's $d = 0.664$, 95%CI [0.36, 0.97]
**Sample**	**Parochial Moral Beliefs and Values**
**Moral Valuation of Ingroup Loyalty (MFQ–Loyalty)**	
Omnibus	$F(2, 357) = 5.35$, $p = .005$, $\eta^2p = .029$, 95%CI [0.00, 0.07]
EA vs. Control	$t(357) = -2.77$, $p = .018$, Cohen's $d = -0.329$, 95%CI [-0.56, -0.09]
XA vs. Control	$t(357) = -2.56$, $p = .033$, Cohen's $d = -0.371$, 95%CI [-0.66, -0.08]
EA vs. XA	$t(357) = 0.27$, $p > .999$, Cohen's $d = 0.042$, 95%CI [-0.26, 0.35]
**Moral Valuation of Familial Loyalty (MAC-Q–Familial Loyalty)**	
Omnibus	$F(2, 357) = 16.30$, $p < .001$, $\eta^2p = .083$, 95%CI [0.03, 0.14]
EA vs. Control	$t(357) = -5.70$, $p < .001$, Cohen's $d = -0.676$, 95%CI [-0.92, -0.44]
XA vs. Control	$t(357) = -1.70$, $p = .269$, Cohen's $d = -0.247$, 95%CI [-0.53, 0.04]
EA vs. XA	$t(357) = -2.78$, $p = .017$, Cohen's $d = -0.429$, 95%CI [-0.73, -0.12]
**Moral Valuation of Group Loyalty (MAC-Q–Group Loyalty)**	
Omnibus	$F(2, 357) = 4.43$, $p = .013$, $\eta^2p = .024$, 95%CI [0.00, 0.06]
EA vs. Control	$t(357) = -0.17$, $p > .999$, Cohen's $d = -0.020$, 95%CI [-0.25, 0.21]
XA vs. Control	$t(357) = 2.75$, $p = .019$, Cohen's $d = 0.399$, 95%CI [0.12, 0.69]
EA vs. XA	$t(357) = -2.72$, $p = .021$, Cohen's $d = -0.419$, 95%CI [-0.72, -0.11]
**Sample**	**Exploratory Moral Beliefs and Values**
**Moral Judgments of Instrumental Harm (OUS–IH)**	
Omnibus	$F(2, 357) = 42.40$, $p < .001$, $\eta^2p = .192$, 95%CI [0.12, 0.26]
EA vs. Control	$t(357) = 8.07$, $p < .001$, Cohen's $d = 0.958$, 95%CI [0.71, 1.20]
XA vs. Control	$t(357) = -1.53$, $p = .379$, Cohen's $d = -0.222$, 95%CI [-0.51, 0.06]
EA vs. XA	$t(357) = 7.65$, $p < .001$, Cohen's $d = 1.180$, 95%CI [0.86, 1.50]
**Moral Valuation of Authority (MFQ–Authority)**	
Omnibus	$F(2, 357) = 8.48$, $p < .001$, $\eta^2p = .045$, 95%CI [0.01, 0.09]
EA vs. Control	$t(357) = -4.03$, $p < .001$, Cohen's $d = -0.479$, 95%CI [-0.71, -0.24]
XA vs. Control	$t(357) = -2.11$, $p = .107$, Cohen's $d = -0.306$, 95%CI [-0.59, -0.02]
EA vs. XA	$t(357) = -1.12$, $p = .791$, Cohen's $d = -0.173$, 95%CI [-0.48, 0.13]
**Moral Valuation of Purity (MFQ–Purity)**	
Omnibus	$F(2, 357) = 13.10$, $p < .001$, $\eta^2p = .068$, 95%CI [0.02, 0.12]
EA vs. Control	$t(357) = -4.49$, $p < .001$, Cohen's $d = -0.533$, 95%CI [-0.77, -0.30]
XA vs. Control	$t(357) = -3.79$, $p < .001$, Cohen's $d = -0.550$, 95%CI [-0.83, -0.26]
EA vs. XA	$t(357) = 0.11$, $p > .999$, Cohen's $d = 0.017$, 95%CI [-0.29, 0.32]
**Moral Valuation of Reciprocity (MAC-Q–Reciprocity)**	

Omnibus	$F(2, 357) = 7.56$, $p < .001$, $\eta^2 p = .041$, 95%CI [0.01, 0.08]
EA vs. Control	$t(357) = -3.72$, $p < .001$, Cohen's $d = -0.442$, 95%CI [-0.67, -0.21]
XA vs. Control	$t(357) = -0.16$, $p > .999$, Cohen's $d = -0.023$, 95%CI [-0.31, 0.26]
EA vs. XA	$t(357) = -2.71$, $p = .021$, Cohen's $d = -0.418$, 95%CI [-0.72, -0.11]
**Moral Valuation of Deference to Authority (MAC-Q–Deference)**	
Omnibus	$F(2, 357) = 5.46$, $p = .005$, $\eta^2 p = .030$, 95%CI [0.00, 0.07]
EA vs. Control	$t(357) = -3.16$, $p = .005$, Cohen's $d = -0.375$, 95%CI [-0.61, -0.14]
XA vs. Control	$t(357) = -1.96$, $p = .153$, Cohen's $d = -0.284$, 95%CI [-0.57, 0.00]
EA vs. XA	$t(357) = -0.59$, $p > .999$, Cohen's $d = -0.091$, 95%CI [-0.39, 0.21]
**Moral Valuation of Heroism (MAC-Q–Heroism)**	
Omnibus	$F(2, 357) = 13.00$, $p < .001$, $\eta^2 p = .068$, 95%CI [0.02, 0.12]
EA vs. Control	$t(357) = -4.85$, $p < .001$, Cohen's $d = -0.576$, 95%CI [-0.81, -0.34]
XA vs. Control	$t(357) = -0.13$, $p > .999$, Cohen's $d = -0.019$, 95%CI [-0.31, 0.27]
EA vs. XA	$t(357) = -3.61$, $p = .001$, Cohen's $d = -0.556$, 95%CI [-0.86, -0.25]
**Moral Valuation of Property Rights (MAC-Q–Property Rights)**	
Omnibus	$F(2, 357) = 10.60$, $p < .001$, $\eta^2 p = .056$, 95%CI [0.02, 0.11]
EA vs. Control	$t(357) = -4.49$, $p < .001$, Cohen's $d = -0.532$, 95%CI [-0.77, -0.30]
XA vs. Control	$t(357) = -0.54$, $p > .999$, Cohen's $d = -0.079$, 95%CI [-0.36, 0.21]
EA vs. XA	$t(357) = -2.94$, $p = .010$, Cohen's $d = -0.454$, 95%CI [-0.76, -0.15]

Sample Differences in Finer-Grained Moral-Circle Distinctions

Table S4. Results from one-way ANOVAs with Bonferroni-corrected post-hoc tests comparing Samples 1-3 on finer-grained distinctions within the moral circle

Sample	Moral Concern
**Moral Concern for Friends and Family Members (MES–Friends & Family)**	
Omnibus	$F(2, 357) = 9.21$, $p < .001$, $\eta^2p = .049$, 95%CI [0.01, 0.10]
EA vs. Control	$t(357) = -3.63$, $p < .001$, Cohen's $d = -0.431$, 95%CI [-0.67, -0.20]
XA vs. Control	$t(357) = 0.96$, $p > .999$, Cohen's $d = 0.139$, 95%CI [-0.15, 0.42]
EA vs. XA	$t(357) = -3.70$, $p < .001$, Cohen's $d = -0.570$, 95%CI [-0.88, -0.26]
**Moral Concern for Ingroups (MES–Ingroup)**	
Omnibus	$F(2, 357) = 20.90$, $p < .001$, $\eta^2p = .105$, 95%CI [0.05, 0.16]
EA vs. Control	$t(357) = 5.75$, $p < .001$, Cohen's $d = 0.683$, 95%CI [0.44, 0.92]
XA vs. Control	$t(357) = 4.67$, $p < .001$, Cohen's $d = 0.678$, 95%CI [0.39, 0.97]
EA vs. XA	$t(357) = 0.03$, $p > .999$, Cohen's $d = 0.005$, 95%CI [-0.30, 0.31]
**Moral Concern for Revered Humans (MES–Revered)**	
Omnibus	$F(2, 357) = 18.10$, $p < .001$, $\eta^2p = .092$, 95%CI [0.04, 0.15]
EA vs. Control	$t(357) = 5.72$, $p < .001$, Cohen's $d = 0.679$, 95%CI [0.44, 0.92]
XA vs. Control	$t(357) = 3.65$, $p < .001$, Cohen's $d = 0.530$, 95%CI [0.24, 0.82]
EA vs. XA	$t(357) = 0.97$, $p > .999$, Cohen's $d = 0.149$, 95%CI [-0.15, 0.45]
**Moral Concern for Stigmatized Humans (MES–Stigmatized)**	
Omnibus	$F(2, 357) = 30.10$, $p < .001$, $\eta^2p = .144$, 95%CI [0.08, 0.21]
EA vs. Control	$t(357) = 6.07$, $p < .001$, Cohen's $d = 0.959$, 95%CI [0.48, 0.96]
XA vs. Control	$t(357) = 6.57$, $p < .001$, Cohen's $d = 1.248$, 95%CI [0.66, 1.25]
EA vs. XA	$t(357) = -1.52$, $p = .390$, Cohen's $d = -0.234$, 95%CI [-0.54, 0.07]
**Moral Concern for Outgroups (MES–Outgroup)**	
Omnibus	$F(2, 357) = 40.20$, $p < .001$, $\eta^2p = .184$, 95%CI [0.11, 0.25]
EA vs. Control	$t(357) = 8.08$, $p < .001$, Cohen's $d =0.959$, 95%CI [0.72, 1.20]
XA vs. Control	$t(357) = 6.33$, $p < .001$, Cohen's $d = 0.919$, 95%CI [0.63, 1.21]
EA vs. XA	$t(357) = 0.26$, $p > .999$, Cohen's $d = 0.040$, 95%CI [-0.26, 0.34]
**Moral Concern for High-Sentience Animals (MES–High-Sentience Animals)**	
Omnibus	$F(2, 357) = 15.10$, $p < .001$, $\eta^2p = .078$, 95%CI [0.03, 0.13]
EA vs. Control	$t(357) = 5.44$, $p < .001$, Cohen's $d =0.645$, 95%CI [0.41, 0.88]
XA vs. Control	$t(357) = 2.57$, $p = .032$, Cohen's $d = 0.373$, 95%CI [0.08, 0.66]
EA vs. XA	$t(357) = 1.77$, $p = .234$, Cohen's $d = 0.273$, 95%CI [-0.03, 0.58]
**Moral Concern for Low-Sentience Animals (MES–Low-Sentience Animals)**	
Omnibus	$F(2, 357) = 18.60$, $p < .001$, $\eta^2p = .094$, 95%CI [0.04, 0.15]
EA vs. Control	$t(357) = 6.10$, $p < .001$, Cohen's $d =0.724$, 95%CI [0.48, 0.96]
XA vs. Control	$t(357) = 2.02$, $p < .001$, Cohen's $d = 0.294$, 95%CI [0.00, 0.58]
EA vs. XA	$t(357) = 2.79$, $p = .017$, Cohen's $d = 0.430$, 95%CI [0.12, 0.74]
**Moral Concern for Plants (MES–Plants)**	
Omnibus	$F(2, 357) = 1.40$, $p = .248$, $\eta^2p = .008$, 95%CI [0.00, 0.03]
EA vs. Control	$t(357) = 1.31$, $p = .572$, Cohen's $d = 0.156$, 95%CI [-0.08, 0.39]
XA vs. Control	$t(357) = 1.42$, $p = .474$, Cohen's $d = 0.205$, 95%CI [-0.08, 0.49]
EA vs. XA	$t(357) = -0.32$, $p > .999$, Cohen's $d = -0.050$, 95%CI [-0.35, 0.25]
**Moral Concern for Natural Environments (MES–Environments)**	
Omnibus	$F(2, 357) = 3.18$, $p = .043$, $\eta^2p = .018$, 95%CI [0.00, 0.05]
EA vs. Control	$t(357) = 1.94$, $p = .160$, Cohen's $d = 0.230$, 95%CI [-0.00, 0.46]
XA vs. Control	$t(357) = 2.16$, $p = .093$, Cohen's $d = 0.314$, 95%CI [0.03, 0.60]
EA vs. XA	$t(357) = -0.55$, $p > .999$, Cohen's $d = -0.084$, 95%CI [-0.39, 0.22]
**Moral Concern for Villains (MES–Villains)**	
Omnibus	$F(2, 357) = 72.0$, $p < .001$, $\eta^2p = .287$, 95%CI [0.21, 0.36]
EA vs. Control	$t(357) = 11.94$, $p < .001$, Cohen's $d = 1.416$, 95%CI [1.16, 1.67]
XA vs. Control	$t(357) = 5.09$, $p < .001$, Cohen's $d = 0.739$, 95%CI [0.45, 1.03]
EA vs. XA	$t(357) = 4.39$, $p < .001$, Cohen's $d = 0.678$, 95%CI [0.37, 0.99]

Regressions Between Measures of Morality and Prosociality

Table S5. Relationships (standardized regression coefficients) between moral beliefs and values with general prosociality and equity/effectiveness in altruism among EAs, XAs, and general population controls

Sample	*Moral Beliefs & Values*	General Prosociality		Effective Altruism		Equitable Altruism	Equitable & Effective Altruism Combined	
EA	***MES***	**RWCA Money**	**RWCA Time**	**EAIS–EF**	**BDT**	**EAIS–EX**	**MJV**	**SDT**
		R^2=.01	R^2=.00	R^2=.08	R^2=.05	R^2=.23	R^2=.08	R^2=.07
	Overall Moral Concern	0.11	-0.06	0.29**	0.22*	0.48***	0.28**	0.27**
	MFQ[1]	**RWCA Money**	**RWCA Time**	**EAIS–EF**	**BDT**	**EAIS–EX**	**MJV**	**SDT**
		R^2=.08	R^2=.27	R^2=.20	R^2=.34	R^2=.23	R^2=.30	R^2=.12
	Harm	-0.16	-0.19	0.28*	0.24*	0.41***	0.28*	0.46***
	Fairness	-0.07	0.19†	-0.03	0.09	0.02	0.09	-0.22†
	Loyalty	-0.13	0.12	-0.22	-0.47**	-0.04	-0.30*	-0.25
	Authority	-0.31	-0.48**	0.51**	0.38*	0.00	0.45*	0.32
	Purity	0.46*	0.78***	-0.66***	-0.48**	-0.31†	-0.64***	-0.13
	MAC-Q[2]	**RWCA Money**	**RWCA Time**	**EAIS–EF**	**BDT**	**EAIS–EX**	**MJV**	**SDT**
		R^2=.12	R^2=.20	R^2=.27	R^2=.31	R^2=.31	R^2=.28	R^2=.16
	Fairness	0.03	0.10	0.15	0.11	0.34***	0.24*	-0.07
	Familial Loyalty	-0.08	-0.23	-0.31*	-0.28*	-0.36**	-0.20	-0.40**
	Group Loyalty	-0.14	0.11	0.33*	0.20	0.41**	0.18	0.51***
	Reciprocity	-0.17	-0.28	-0.28*	0.06	-0.39**	-0.01	0.00
	Deference	0.36*	0.48**	-0.45**	-0.61***	-0.48***	-0.56***	0.05
	Heroism	0.05	0.19	0.10	0.10	0.31†	0.11	-0.12
	Property Rights	-0.24*	-0.03	0.40***	0.21*	0.18†	0.28**	0.23*
	OUS[3]	**RWCA Money**	**RWCA Time**	**EAIS–EF**	**BDT**	**EAIS–EX**	**MJV**	**SDT**
		R^2=.00	R^2=.02	R^2=.23	R^2=.03	R^2=.27	R^2=.12	R^2=.08
	Impartial Beneficence	0.05	0.10	0.34***	0.16	0.50***	0.30**	0.26*
	Instrumental Harm	-0.05	-0.17†	0.21*	0.04	0.04	0.08	0.03
XA	***MES***	**RWCA Money**	**RWCA Time**	**EAIS–EF**	**BDT**	**EAIS–EX**	**MJV**	**SDT**
		R^2=.02	R^2=.00	R^2=.00	R^2=.05	R^2=.08	R^2=.00	R^2=.02
	Overall Moral Concern	-0.14	-0.04	0.02	0.23†	0.29*	-0.07	0.14
	MFQ[1]	**RWCA Money**	**RWCA Time**	**EAIS–EF**	**BDT**	**EAIS–EX**	**MJV**	**SDT**
		R^2=.02	R^2=.11	R^2=.11	R^2=.18	R^2=.20	R^2=.21	R^2=.07
	Harm	0.07	-0.29	-0.06	0.16	0.26	0.16	-0.17
	Fairness	-0.02	0.14	0.09	0.11	0.12	-0.24	0.07
	Loyalty	0.21	-0.15	0.20	-0.36†	-0.32	-0.08	-0.11
	Authority	-0.11	0.33	0.14	0.19	0.02	-0.02	0.14
	Purity	-0.10	0.08	-0.43*	-0.21	0.34*	-0.37*	0.25
	MAC-Q[2]	**RWCA Money**	**RWCA Time**	**EAIS–EF**	**BDT**	**EAIS–EX**	**MJV**	**SDT**
		R^2=.07	R^2=.12	R^2=.05	R^2=.20	R^2=.18	R^2=.17	R^2=.09
	Fairness	-0.08	0.15	0.23	0.03	-0.01	-0.27	-0.07
	Familial Loyalty	0.05	-0.37†	0.18	-0.13	0.03	-0.18	-0.27
	Group Loyalty	0.03	0.33	-0.00	0.37†	0.60**	0.07	0.21
	Reciprocity	0.02	-0.05	-0.05	0.20	-0.16	0.35†	0.16
	Deference	-0.16	0.30	-0.15	-0.41*	-0.02	-0.41*	0.34†
	Heroism	0.27	-0.09	0.06	-0.11	-0.23	0.19	-0.26
	Property Rights	0.10	-0.07	-0.15	-0.00	-0.00	0.08	0.02
	OUS[3]	**RWCA Money**	**RWCA Time**	**EAIS–EF**	**BDT**	**EAIS–EX**	**MJV**	**SDT**
		R^2=.03	R^2=.04	R^2=.11	R^2=.05	R^2=.12	R^2=.17	R^2=.03
	Impartial Beneficence	0.09	-0.17	0.03	0.19	0.34**	0.08	0.13
	Instrumental Harm	-0.15	-0.09	0.32**	0.12	-0.01	0.06	0.13
Control	***MES***	**RWCA Money**	**RWCA Time**	**EAIS–EF**	**BDT**	**EAIS–EX**	**MJV**	**SDT**
		R^2=.02	R^2=.00	R^2=.00	R^2=.00	R^2=.05	R^2=.01	R^2=.03
	Overall Moral Concern	0.14†	0.03	-0.03	0.03	0.22**	0.11	0.16*
	MFQ[1]	**RWCA Money**	**RWCA Time**	**EAIS–EF**	**BDT**	**EAIS–EX**	**MJV**	**SDT**
		R^2=.09	R^2=.09	R^2=.10	R^2=.14	R^2=.19	R^2=.08	R^2=.07
	Harm	0.16†	0.09	-0.18†	0.09	0.29**	-0.04	0.14
	Fairness	-0.10	0.06	0.17†	0.24**	0.13	0.21*	-0.01
	Loyalty	0.15	0.02	0.31**	-0.08	0.10	-0.10	0.02
	Authority	-0.12	0.19	0.07	-0.14	-0.11	0.08	-0.05
	Purity	0.19	0.05	-0.05	-0.09	0.14	-0.20†	0.20†
	MAC-Q[2]	**RWCA Money**	**RWCA Time**	**EAIS–EF**	**BDT**	**EAIS–EX**	**MJV**	**SDT**
		R^2=.11	R^2=.09	R^2=.06	R^2=.17	R^2=.23	R^2=.13	R^2=.05
	Fairness	-0.03	-0.00	-0.05	0.31***	0.20*	0.19*	-0.12
	Familial Loyalty	-0.02	0.32*	0.07	-0.10	-0.09	-0.34**	0.08

Group Loyalty	0.24*	0.15	0.03	0.24*	0.47***	0.18†	0.10
Reciprocity	-0.09	-0.13	0.03	-0.12	-0.13	-0.18	0.07
Deference	0.18	-0.03	0.25*	-0.21*	0.06	-0.02	0.00
Heroism	0.05	-0.02	-0.04	-0.17	-0.01	0.05	0.05
Property Rights	-0.06	-0.06	-0.18†	0.04	-0.12	-0.00	0.01
***OUS*[3]**	**RWCA Money**	**RWCA Time**	**EAIS–EF**	**BDT**	**EAIS–EX**	**MJV**	**SDT**
	R^2=.07	R^2=.03	R^2=.18	R^2=.01	R^2=.26	R^2=.00	R^2=.07
Impartial Beneficence	0.26*	0.11	0.24***	0.09	0.51***	-0.02	0.25***
Instrumental Harm	-0.09	-0.13†	0.34***	-0.10	-0.08	-0.01	-0.07

Note. †*p* < .10, **p* < .05, ***p* <.01, ****p* < .001. [1]Includes all MFQ subscales as simultaneous predictors. [2]Includes all MAC-Q subscales as simultaneous predictors. [3]Includes all OUS subscales as simultaneous predictors.

Bivariate Correlations Between Measures of Morality and Prosociality

Table S6. Relationships between moral beliefs and values with general prosociality and equity/effectiveness in altruism among EAs, XAs, and general population controls

Moral Beliefs & Values	**General Prosociality**		**Effective Altruism**		**Equitable Altruism**	**Equitable & Effective Altruism Combined**	
EAs	RWCA Money	RWCA Time	EAIS–EF	BDT	EAIS–EX	MJV	SDT
Overall Moral Concern (MES)	0.11	-0.06	0.29**	0.22*	0.48***	0.28**	0.27**
Harm (MFQ)	-0.16	0.10	0.11	0.15	0.35***	0.19*	0.26**
Fairness (MFQ)	-0.15	0.17	0.07	0.15	0.26**	0.18	0.07
Loyalty (MFQ)	-0.05	0.34***	-0.28**	-0.48***	-0.21*	-0.37***	-0.03
Authority (MFQ)	-0.03	0.28**	-0.22*	-0.39***	-0.27**	-0.32***	0.04
Purity (MFQ)	0.05	0.45***	-0.34***	-0.46***	-0.25**	-0.42***	0.03
Fairness (MAC-Q)	-0.11	0.11	0.09	0.08	0.24**	0.23*	0.09
Familial Loyalty (MAC-Q)	-0.08	0.19*	-0.24**	-0.33***	-0.22*	-0.18	-0.01
Group Loyalty (MAC-Q)	-0.14	0.20*	-0.05	-0.10	0.05	0.01	0.25**
Reciprocity (MAC-Q)	-0.18*	0.09	-0.12	-0.05	-0.09	0.05	0.16
Deference (MAC-Q)	0.06	0.38***	-0.31***	-0.45***	-0.29**	-0.31***	0.06
Heroism (MAC-Q)	-0.05	0.29**	-0.20*	-0.26**	-0.12	-0.13	0.06
Property Rights (MAC-Q)	-0.20*	0.11	0.11	-0.05	-0.03	0.09	0.16
Impartial Beneficence (OUS)	0.03	0.02	0.44***	0.18*	0.52***	0.34***	0.28**
Instrumental Harm (OUS)	-0.03	-0.13	0.37***	0.12	0.27**	0.22*	0.16
**XAs**	RWCA Money	RWCA Time	EAIS–EF	BDT	EAIS–EX	MJV	SDT
Overall Moral Concern (MES)	-0.14	-0.04	0.02	0.23	0.29*	-0.07	0.14
Harm (MFQ)	0.06	-0.13	-0.03	0.14	0.35**	-0.12	-0.05
Fairness (MFQ)	0.03	0.00	0.08	0.20	0.26*	-0.17	-0.02
Loyalty (MFQ)	0.07	0.11	0.03	-0.30*	0.00	-0.34**	0.13
Authority (MFQ)	0.00	0.23	0.03	-0.16	0.07	-0.33**	0.19
Purity (MFQ)	-0.01	0.12	-0.21	-0.28*	0.21	-0.41***	0.22
Fairness (MAC-Q)	0.06	0.15	0.17	0.09	0.10	-0.14	0.04
Familial Loyalty (MAC-Q)	0.16	0.01	0.10	-0.11	0.16	-0.17	0.03
Group Loyalty (MAC-Q)	0.17	0.20	0.09	0.13	0.35**	-0.01	0.13
Reciprocity (MAC-Q)	0.14	0.11	0.09	0.08	0.11	0.04	0.13
Deference (MAC-Q)	0.09	0.15	0.02	-0.26*	0.08	-0.24	0.16
Heroism (MAC-Q)	0.23	0.09	0.07	-0.10	0.06	-0.01	0.01
Property Rights (MAC-Q)	0.14	0.01	0.01	-0.01	0.10	-0.06	0.03
Impartial Beneficence (OUS)	0.09	-0.17	0.02	0.19	0.34**	0.08	0.12
Instrumental Harm (OUS)	-0.15	-0.09	0.32**	0.11	-0.02	0.06	0.13
**Controls**	RWCA Money	RWCA Time	EAIS–EF	BDT	EAIS–EX	MJV	SDT
Overall Moral Concern (MES)	0.14	0.03	-0.03	0.03	0.22**	0.11	0.16*
Harm (MFQ)	0.19*	0.20**	0.03	0.13	0.40***	0.00	0.19*
Fairness (MFQ)	0.01	0.13	0.10	0.27***	0.29***	0.18*	0.07
Loyalty (MFQ)	0.24**	0.22**	0.28***	-0.19*	0.24**	-0.17*	0.18*
Authority (MFQ)	0.17*	0.26***	0.22**	-0.22**	0.16*	-0.14	0.16*
Purity (MFQ)	0.25**	0.23**	0.17*	-0.20**	0.23**	-0.21**	0.23**
Fairness (MAC-Q)	0.06	0.04	0.01	0.22**	0.27***	0.09	-0.01
Familial Loyalty (MAC-Q)	0.19*	0.27***	0.14	-0.18*	0.13	-0.28***	0.19*
Group Loyalty (MAC-Q)	0.28***	0.22**	0.14	0.02	0.41***	-0.05	0.18*
Reciprocity (MAC-Q)	0.13	0.10	0.09	-0.06	0.18*	-0.16*	0.15*
Deference (MAC-Q)	0.25***	0.15*	0.20**	-0.23**	0.16*	-0.17*	0.15*
Heroism (MAC-Q)	0.23**	0.17*	0.13	-0.17*	0.21**	-0.13	0.17*
Property Rights (MAC-Q)	0.05	0.05	-0.02	-0.11	-0.06	-0.16*	0.10
Impartial Beneficence (OUS)	0.25***	0.10	0.26***	0.09	0.51***	-0.02	0.25***
Instrumental Harm (OUS)	-0.08	-0.13	0.35***	-0.10	-0.06	-0.01	-0.06

Note. *p < .05, **p <.01, ***p < .001. Bolded values denote statistically significant associations.

Additional Detail on Measures of Moral Beliefs and Values

The 32-item Moral Foundations Questionnaire (MFQ[1]) measured participants' endorsement of five moral values: care (harm reduction), fairness, loyalty, authority, and purity. According to Moral Foundations Theory (MFT), care and fairness ("individualizing" foundations) promote personal freedom and are broadly endorsed, while loyalty, authority, and purity ("binding" foundations) support group cohesion and are more common among conservatives[2,3]. Notably, predominant theoretical perspectives associate the value of loyalty with ingroup favoritism[4]. Similarly, the 42-item Morality as Cooperation Questionnaire (MAC-Q[5]) assessed the importance of seven evolutionarily-informed moral values derived from Morality as Cooperation Theory (MAC-T): familial loyalty, group loyalty, reciprocity, heroism, deference, fairness, and property. These values largely align with those from MFQ but distinguish kin-based from group-based loyalty to account for kin-selection and group-selection, respectively—two distinct adaptive mechanisms central to biological models of cooperation[6,7]. Moreover, MAC-T additionally acknowledges heroism and property rights as discrete values within the human moral taxonomy and, unlike MFT, does not include a value for purity.

The Oxford Utilitarianism Scale[8] captured moral beliefs in line with two dimensions of utilitarianism: impartial beneficence (i.e., the *positive* dimension of utilitarianism—prioritizing prosociality towards recipients that stand to benefit the most, even if those beneficiaries are socially distant; e.g., "It is morally wrong to keep money that one doesn't really need if one can donate it to causes that provide effective help to those who will benefit a great deal.") and instrumental harm (i.e., the negative dimension of utilitarian—permitting harm for the greater good; e.g., "It is permissible to torture an innocent person if this would be necessary to provide information to prevent a bomb going off that would kill hundreds of people.").

Finally, the Moral Expansiveness Scale (MES; Crimston et al., 2016) measured the breadth and depth of participants' "circles" of moral concern. Participants placed a series of human and non-human entities at varying degrees of social distance within four boundaries of moral regard, placing the entities to whom they ascribe greater moral value/feel the most obligated to protect within the inner-most circles, while placing the entities to whom they ascribe comparatively lesser moral value/feel less obligation to protect within the outer-most circles. Typically, people place socially closer entities (e.g., family members,

friends, ingroups) within the inner-most circles and socially-distant humans (e.g., human outgroups, stigmatized people) and non-human entities in the outer-most circles[9]. To capture overall moral concern, scores on the MES were aggregated across all entities on the scale for each participant, with higher scores representing more expansive moral circles. Additionally, to capture moral concern in a more fine-grained manner, separate composites were calculated across 10 entity categories, each comprising three entities for a total of 30 items: family/friends (e.g., a partner or spouse), ingroup humans (e.g., someone from the same country), revered humans (e.g., a charity worker), stigmatized humans (e.g., a mentally-challenged individual), human outgroups (e.g., someone from a different country), high-sentience animals (e.g., a dolphin), low-sentience animals (e.g., a bee), plants (e.g., a tree), environments (e.g., a coral reef), and villains (e.g., a murder).

Moderation Analysis

Table S7. Examining whether moral expansiveness moderates the relationship between group loyalty and expansive altruism on the EAIS-EX

			95% Confidence Interval			
	Estimate	SE	Lower	Upper	Z	p
**EA**						
MAC-Group	0.05	0.004	-0.004	0.013	1.024	.306
MES	1.118	0.187	0.75	1.485	5.974	< .001
MAC-Group ✻ MES	-0.001	0.008	-0.018	0.015	-0.137	.891
**XA**						
MAC-Group	0.019	0.007	0.005	0.033	2.657	.008
MES	0.457	0.237	-0.008	0.922	1.928	.054
MAC-Group ✻ MES	0.010	0.014	-0.0179	0.037	0.690	.490
**Control**						
MAC-Group	0.023	0.004	0.016	0.031	5.912	< .001
MES	0.472	0.163	0.154	0.792	2.903	.004
MAC-Group ✻ MES	-0.003	0.008	-0.018	0.012	-0.340	.734

Figure S1. The relationship between group loyalty and expansive altruism on the EAIS-EX at different levels of overall moral concern on the MES among XAs

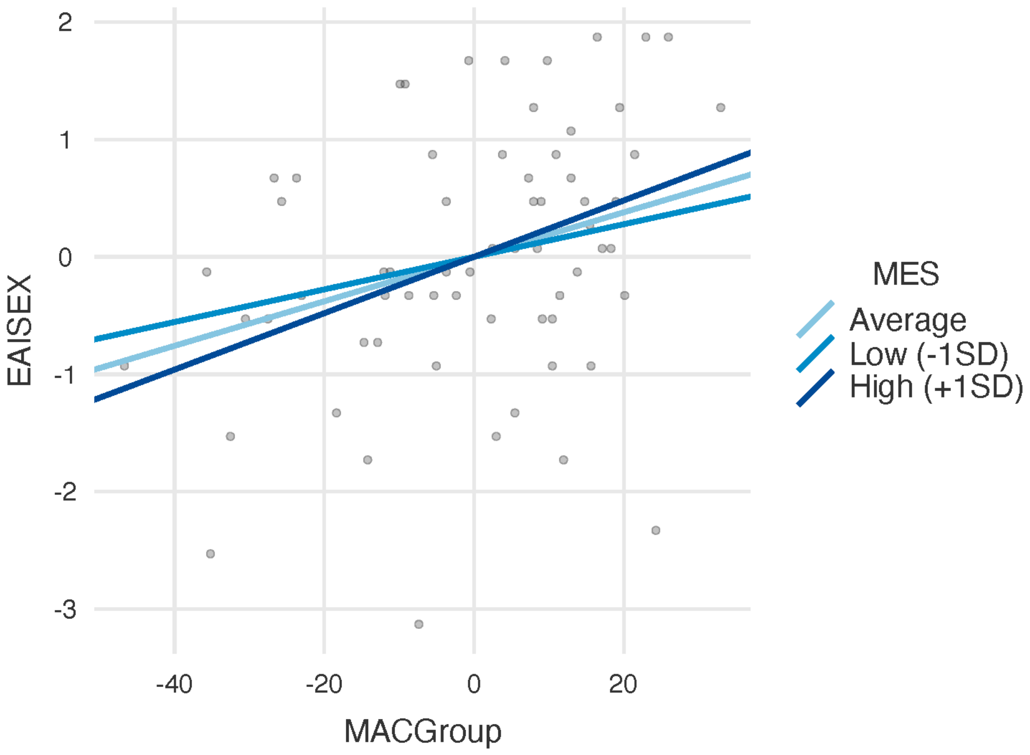

Supplementary References

1. Graham, J. *et al.* Mapping the Moral Domain. *J Pers Soc Psychol* 101, 366–385 (2011).
2. Day, M. V., Fiske, S. T., Downing, E. L. & Trail, T. E. Shifting liberal and conservative attitudes using moral foundations theory. *Pers Soc Psychol Bull* 40, 1559–1573 (2014).
3. Waytz, A., Dungan, J. & Young, L. The whistleblower's dilemma and the fairness–loyalty tradeoff. *Journal of Experimental Social Psychology* 49, 1027–1033 (2013).
4. Graham, J., Waytz, A., Meindl, P., Iyer, R. & Young, L. Centripetal and centrifugal forces in the moral circle: Competing constraints on moral learning. *Cognition* 167, 58–65 (2017).
5. Curry, O., Mullins, D. & Whitehouse, H. Is it good to cooperate? Testing the theory of morality-as-cooperation in 60 societies. *Current Anthropology* 60, (2019).
6. Hamilton, W. D. The genetical evolution of social behaviour. I. *Journal of Theoretical Biology* 7, 1–16 (1964).
7. Trivers, R. L. The Evolution of Reciprocal Altruism. *The Quarterly Review of Biology* 46, 35–57 (1971).
8. Kahane, G. *et al.* Beyond sacrificial harm: A two-dimensional model of utilitarian psychology. *Psychol Rev* 125, 131–164 (2018).
9. Crimston, C., Hornsey, M. J., Bain, P. G. & Bastian, B. Toward a Psychology of Moral Expansiveness. *Current Directions in Psychological Science* 27, 14–19 (2018).

Survey Questions

The measures of moral beliefs and values and the prosociality metrics were presented in a randomized order. Basic demographic items were collected at the end of the survey. Items within the measures were also presented in a randomized order.

Measures of Moral Beliefs and Values

Moral Foundations Questionnaire (Graham et al., 2014)

Part 1. When you decide whether something is right or wrong, to what extent are the following considerations relevant to your thinking? Please rate each statement using this scale:

0- not at all relevant (This consideration has nothing to do with my judgments of right and wrong)	1- not very relevant	2- slightly relevant	3-somewhat relevant	4- very relevant	5- extremely relevant (This is one of the most important factors when I judge right and wrong)

Whether or not someone suffered emotionally
Whether or not some people were treated differently than others
Whether or not someone's action showed love for his or her country
Whether or not someone showed a lack of respect for authority
Whether or not someone violated standards of purity and decency
Whether or not someone was good at math
Whether or not someone cared for someone weak or vulnerable
Whether or not someone acted unfairly
Whether or not someone did something to betray his or her group
Whether or not someone conformed to the traditions of society
Whether or not someone did something disgusting
Whether or not someone was cruel
Whether or not someone was denied his or her rights
Whether or not someone showed a lack of loyalty
Whether or not an action caused chaos or disorder
Whether or not someone acted in a way that God would approve of

Part 2. Please read the following sentences and indicate your agreement or disagreement:

Strongly disagree	Moderately disagree	Slightly disagree	Slightly agree	Moderately agree	Strongly agree

Compassion for those who are suffering is the most crucial virtue.
When the government makes laws, the number one principle should be ensuring that everyone is treated fairly.
I am proud of my country's history.
Respect for authority is something all children need to learn.
People should not do things that are disgusting, even if no one is harmed.
It is better to do good than to do bad.
One of the worst things a person could do is hurt a defenseless animal.
Justice is the most important requirement for a society.
People should be loyal to their family members, even when they have done something wrong.

Men and women each have different roles to play in society.
I would call some acts wrong on the grounds that they are unnatural.
It can never be right to kill a human being.
I think it's morally wrong that rich children inherit a lot of money while poor children inherit nothing.
It is more important to be a team player than to express oneself.
If I were a soldier and disagreed with my commanding officer's orders, I would obey anyway because that is my duty.
Chastity is an important and valuable virtue.

Moral Expansiveness Scale (Crimston et al., 2016)

People sometimes talk about 'circles of moral concern'. These circles are simple ways to make sense of the levels of moral consideration I have for different entities (e.g., people, animals, and the environment). Where I place these entities within our moral circles is important as it reflects their moral worth, and has direct consequences for how I treat them.
On the following page you are given the opportunity to organise a range of entities and place them within your own moral circles that reflect your individual views and feelings.

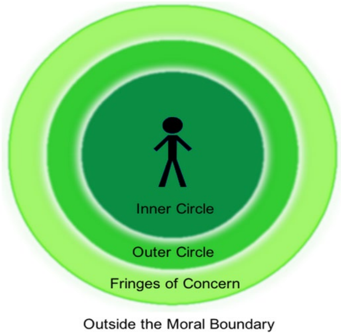

Please read the four boundary descriptions below carefully before completing the moral circle task.

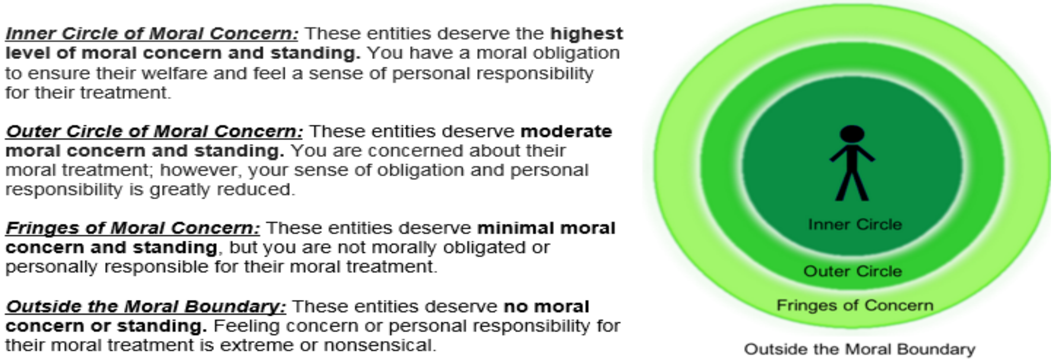

Having carefully read these descriptions, please consider the level of moral concern you personally have for each of the entities below and drop each one into the appropriate moral circle box on the right.

Outside the Moral Boundary	Fringes of Moral Concern	Outer Circle of Moral Concern	Inner Circle of Moral Concern

Somebody from your neighborhood
Homosexual
Bee
Close friend
Partner/spouse
Foreign citizen
Head of State for Your Country (Position Not Specific Person)
Grand Canyon National Park

Chimpanzee
Co-worker
Murderer
Member of opposing political party
Coral reef
Family member
Old-growth forest
Apple tree
Terrorist
Dolphin
Somebody with different religious beliefs
Charity worker
Chicken
Soldier from Your Country
Redwood tree
Refugee
Rose bush
Fish
Mentally challenged individual
Child molester
Citizen of Your Country
Cow

Morality as Cooperation Questionnaire (Curry et al., 2019)

Relevance Items
When you decide whether something is right or wrong, to what extent are the following considerations relevant to your thinking? (0–100; not at all relevant, not very relevant, slightly relevant, somewhat relevant, very relevant, extremely relevant):
Family
Whether or not someone acted to protect their family.
Whether or not someone helped a member of their family.
Whether or not someone's action showed love for their family.
Group
Whether or not someone acted in a way that helped their community.
Whether or not someone helped a member of their community.
Whether or not someone worked to unite a community.
Reciprocity
Whether or not someone did what they had agreed to do.
Whether or not someone kept their promise.
Whether or not someone proved that they could be trusted.
Heroism
Whether or not someone acted heroically.
Whether or not someone showed courage in the face of adversity.
Whether or not someone was brave.
Deference
Whether or not someone deferred to those in authority.
Whether or not someone disobeyed orders.
Whether or not someone showed respect for authority.
Fairness
Whether or not someone kept the best part for themselves.
Whether or not someone showed favouritism.
Whether or not someone took more than others.
Property
Whether or not someone vandalised another person's property.
Whether or not someone kept something that didn't belong to them.

Whether or not someone's property was damaged.

Judgement Items
To what extent do you agree with the following statements? (0–100; strongly disagree, disagree, neither agree or disagree, agree, strongly agree):
Family:
People should be willing to do anything to help a member of their family.
You should always be loyal to your family.
You should always put the interests of your family first.
Group:
People have an obligation to help members of their community.
It's important for individuals to play an active role in their communities.
You should try to be a useful member of society.
Reciprocity:
You have an obligation to help those who have helped you.
You should always make amends for the things you have done wrong.
You should always return a favour if you can.
Heroism:
Courage in the face of adversity is the most admirable trait.
Society should do more to honour its heroes.
To be willing to lay down your life for your country is the height of bravery.
Deference:
People should always defer to their superiors.
Society would be better if people were more obedient to authority.
You should respect people who are older than you.
Fairness:
Everyone should be treated the same.
Everyone's rights are equally important.
The current levels of inequality in society are unfair.
Property:
It's acceptable to steal food if you are starving. (R)
It's ok to keep valuable items that you find, rather than try to locate the rightful owner. (R)
Sometimes you are entitled to take things you need from other people. (R)

Oxford Utilitarianism Scale (Kahane et al., 2018)

Please rate how much you agree or disagree with each statement:
7-point Likert scale: 1 - Strongly Disagree, 7 - Strongly Agree
1. If the only way to save another person's life during an emergency is to sacrifice one's own leg, then one is morally required to make this sacrifice.
2. It is morally right to harm an innocent person if harming them is a necessary means to helping several other innocent people.
3. From a moral point of view, I should feel obliged to give one of our kidneys to a person with kidney failure since I don't need two kidneys to survive, but really only one to be healthy.
4. If the only way to ensure the overall well-being and happiness of the people is through the use of political oppression for a short, limited period, then political oppression should be used.
5. From a moral perspective, people should care about the well-being of all human beings on the planet equally; they should not favor the well-being of people who are especially close to them either physically or emotionally.
6. It is permissible to torture an innocent person if this would be necessary to provide information to prevent a bomb going off that would kill hundreds of people.
7. It is just as wrong to fail to help someone as it is to actively harm them yourself.
8. Sometimes it is morally necessary for innocent people to die as collateral damage—if more people are saved overall.
9. It is morally wrong to keep money that one doesn't really need if one can donate it to causes that provide effective help to those who will benefit a great deal.

Note. Mean scores on both subscales should be computed. Impartial Beneficence-items 1,3,5,7 and 9; Instrumental Harm-items 2,4,6 and 8.

Prosociality Metrics

Moral Judgment Vignettes (Law et al., 2022)
This portion of the study looks at peoples' reactions to different stories about decision-making. You will read stories of situations where someone needs to decide between multiple options and winds up choosing one. After reading each story, you will be asked to report how morally acceptable or unacceptable you find the decision made by the actor in the passage and explain your reasoning for this judgment.
It's extremely important that you engage with each task as instructed on each trial, because you will need to answer questions about each scenario you read. Focus on engaging with each task as you are instructed, and you'll be fine. Also, please note that experimenters will monitor your responses at the conclusion of the study to ensure you complied with task instructions.
Please read the following stories carefully and answer the questions truthfully.

Please indicate the extent to which you find the action in the story morally acceptable:
1 (completely unacceptable) 2 3 4 5 6 7 8 9 (completely acceptable)

This person wishes to donate some money to a cause. One option is to donate money to a charitable organization which is able to provide (a fellow American, a person from the donor's town, a friend of the donor, a family member of the donor) who is experiencing conditions of famine with food for a month with a $300 donation. Another option is a charitable organization which is able to provide 3 African people living under conditions of famine with food for a month with a $300 donation. The potential donor is unsure of what to do with their money. Eventually, they decide to donate $300 to the charity that can feed 3 African people for $300. To what extent was it morally acceptable for the person in the story to donate money to 3 African people instead of 1 (friend, family member, person from their own country; person from their own town)?

This person recently won a $1,000,000 jackpot from the lottery and wants to donate $100,000 to a cause. One option is to give this money to a hospital, allowing (a person from the same country as the donor, a person from the same town as the donor, a friend of the donor, a family member of the donor) to have a special surgery. While one of these surgeries costs $100,000 to perform, it would save the life of the donor's (compartriot, community member, friend, family member) from a deadly disease. The other option is to give the money to the United Nations to help fight measles and rubella, diseases which plague much of the developing world. It costs $80 to save one life from measles or rubella, so a $100,000 donation would provide enough vaccines to save the lives of 1,250 people from the deadly diseases. The person contemplates where they should donate. Eventually, they decide to donate the $100,000 to the UN charity that can help many people in developing nations. To what extent was it morally acceptable for the person in the story to donate money to the UN charity for people in another country instead of 1 (friend, family member, person from their own country; person from their own town)?

Social Discounting Task (Tuen et al., 2023)
Imagine generating a list of the 100 people closest to you in the world. Number 1 would be your closest friend or relative and number 100 would be a distant acquaintance. Please abstain from listing financial benefactors (e.g., parents, grandparents, spouse).
Please provide the first name and last initial of the person occupying (spot 1, spot 2, spot 4, spot 10, spot 15):
Note. Participants then make a series of 27 choices between a smaller amount of money for themselves and a larger amount for others, using piped text to include the names of the people they listed in part one of the task described above. Please see below for an example:

Would you prefer $25 for yourself or $55 for (name of the person at spot 1)?

Behavioral Donation Task (Developed by Researchers)

You have been selected to help choose which charity to allocate a $1 donation towards. You will now be shown a series of choices where you will be asked to decide which of two different charities you would like the dollar to be donated to. The experimenter will donate the dollar to a charity you select on one of the following trials.

Note: Each charity from the lettered list (i.e., List I) is paired with each charity from the numbered list (i.e., List II) for a total of 16 trials. The number of decisions each participant makes to allocate the dollar towards charities in List II is subtracted from the number of decisions each participant makes to allocate the dollar towards charities in List I, yielding a composite “Effectiveness Index”. The Effectiveness Index ranges from -16 (all ineffective charities) to +16 (all effective charities), with a score of ‘0’ indicating 8 effective decisions and 8 ineffective decisions.

List I: Most Effective (GiveWell, 2022):

A. Malaria Consortium: Providing Medicine to Prevent Malaria in sub-Saharan Africa

Malaria is a deadly disease rampant in sub-Saharan Africa. Seasonal malaria chemoprevention is preventive medicine that saves children’s lives. It is given during the four months of the year when malaria infection rates are especially high. Malaria Consortium saves lives in sub-Saharan Africa by funding the administration of this medication.

B. Against Malaria Foundation: Providing Nets to Prevent Malaria in sub-Saharan Africa

Malaria is a deadly disease rampant in sub-Saharan Africa. Bed nets save lives. Participants hang the nets and sleep under them so they are not bitten by malaria-carrying mosquitoes. The Against Malaria Foundation saves lives in sub-Saharan Africa by funding the provision of bed nets.

C. Hellen Keller International: Providing Supplements to Prevent Vitamin A Deficiency Internationally

Vitamin A deficiency leaves children vulnerable to infections and often leads to death. Vitamin A supplements can restore vitamin A to healthy levels. Hellen Keller International saves lives internationally by providing vitamin A supplements to children under 5 years old.

D. New Incentives: Providing Cash Incentives for Routine Childhood Vaccines in Nigeria

In Nigeria, many infants do not receive all of their recommended vaccines. Vaccines reduce the transmission of deadly, preventable illnesses. New Incentives saves lives in Nigeria by providing cash transfers to incentivize caregivers to bring babies to clinics for routine childhood vaccinations.

List II: Less Effective (Consumer reports, charity navigator):

1. Make-A-Wish America: Granting Wishes to Sick Children in America

Make-A-Wish America grants wishes to American children under the age of 18 with life threatening medical conditions to enrich the human experience with hope, strength and joy. Wishes typically fall into one of four categories: to go on a trip, to have something, to meet a celebrity or to be someone (a policeman, astronaut, actor, etc.).

2. Childhood Leukemia Foundation: Educating and Empowering American Childhood Cancer Patients

Childhood Leukemia Foundation’s programs educate and empower childhood cancer patients in America. The organization primarily provides educational binders to parents of children with cancer, wigs to children suffering from cancer-treatment-related hair loss, and educational wish baskets containing toys, games, and iPads to American childhood cancer patients.

3. Help Heal Veterans: Enriching the Lives of American Veterans with Arts and Crafts

Help Heal Veterans offers a variety of therapeutic craft kits free of charge to America’s veterans, both in-home and at community craft centers. The kits use recycled and sustainable materials, promote healing and show American veterans that they are remembered and cared about.

4. National Caregiving Foundation: Using Mailings to Educate the American Pubic About Alzheimer’s Disease

The National Caregiving Foundation uses direct mail to communicate to the American caregiving community. They use their mailings to educate the American public about Alzheimer disease, including warning signs and symptoms and offer suggestions to caregivers, including care for wounded soldiers.

Effective Altruism Interest Scale (Caviola et al., 2022)

1 (strongly disagree) 2 3 4 (neither agree nor disagree) 5 6 7 (strongly agree)

Effectiveness Focus.
Imagine a situation where you intend to do good (e.g., to improve others' lives or the world) with a certain limited amount of resources available (e.g., your time or money). You can decide how to allocate your resources by choosing from different options that all do good. The stakes are high. In such a situation, when you can choose between different options of doing good...

It would be wrong to do something that only does some amount of good if there is an alternative course of action that would do much more good.
It would be the right choice to refrain from helping one person if that makes it possible to help a larger number of people.
Helping one person is less valuable than helping two people to the same extent.
You should follow evidence and reason to do what is most effective, even if you emotionally prefer another option.
The most important consideration is effectiveness - choosing the option that does the most good per resource invested.
You should usually help a large group of people over a smaller group, even if it seems unfair.

Expansive Altruism.
To what extent do you agree or disagree with the following statements?

As long as my and my family's basic material needs are covered, I want to use a significant amount of my resources (e.g., money or time) to improve the world.
I am willing to make significant sacrifices for people in need that I don't know and will never meet.
People in wealthy countries should donate a substantial proportion of their income to make the world a better place.
I would make a career change if it meant that I could improve the lives of people in need.
We should put a lot of emphasis on the well-being of people who live today.
From a moral perspective, the suffering of all beings matters roughly the same, no matter what species they belong to.

Real-World Charitable Action (Income Donated and Time Volunteering; Developed by Researchers)
In a given year, what percentage (out of 100) of your yearly income do you donate to charity?
In a given year, what percentage (out of 100) of your time do you devote towards volunteering to help others?

Demographics
What is your age in years?
What is your nationality (e.g., United States)?
What is your native language (e.g., English)?
What is your Gender?: female, male, other
How would you describe your race/ethnicity? Please select all that apply: White/Caucasian, Black/African American American, Hispanic/Latino/Latina, Asian/Asian American, Native American, Pacific Islander, Middle Eastern, Multiracial, Other (please specify)
What is your political orientation?: Very liberal (left wing), Liberal (left wing), Somewhat liberal (left wing), Moderate/middle of the road, Somewhat conservative (right wing), Conservative (right wing), Very conservative (right wing)
What is the highest degree or level of school you have completed? If currently enrolled, highest degree received.: No schooling completed, Nursery school to 8th grade, Some high school (no diploma), High school graduate (diploma or the equivalent; e.g., GED), Some college credit (no degree), Trade/Technical/Vocational Training, Associate Degree, Bachelor's Degree, Master's Degree, Professional Degree, Doctorate Degree
What was your total household income (before taxes) during the past 12 months?

How many children under 18 live with you?
What best describes your employment status over the last three months?: Working full-time, Working part-time, Unemployed and looking for work, A homemaker or stay-at-home parent, Student, Retired, Other
What is your current marital status?: Married, Living with a partner, Widowed, Divorced/Separated, Never been married
How many people live or stay in your household at least half the time?
What is your religion?: No religion/Atheist, Unsure/Agnostic, Christian (all denominations), Buddhist, Hindu, Jewish, Muslim, Sikh, Any other religion (please specify)
How devout would you describe your religiosity?: Far below average, Somewhat below average, Average, Somewhat above average, Far above average

Note. As mentioned in the Materials and Methods, additional metrics beyond the scope of the present investigation, but part of the larger project, were also captured in the same survey. These included: Future-Oriented Measures (i.e., Dual Legacy Motives Scale, Longtermism Beliefs Scale, Responsibility to Future Generations Scale); Other-Inclusive Identity and Social Attitude Measures (i.e., Identification with All Humanity Scale, Compassionate Love for Humanity Scale, The Self-Construal Scale, The Self-Other Four Immeasurables Scale, Individual Differences in Anthropomorphism Scale, Blatant Dehumanization Scale, The Allo-Inclusive Identity Scale); Empathic Ability Measures (i.e., Theories of Empathy Scale, Parochial Empathy Scale, Interpersonal Reactivity Index Empathic Concern and Personal Distress Subscales, Emotionally Evocative Statements Task, Toronto Alexithymia Scale, Levenson Self-Report Psychopathy Scale); Reasoning Ability Measures (i.e., Cognitive Reflection Test, Need for Cognition Scale, Rational Experiential Index Rational Ability Subscale, Actively Open-Minded Thinking Scale, Heuristics and Bias Tasks); and Close Relationship Quality Measures (UCLA Lonelieness Scale, Relationship Satisfaction Scale, Investment Model Scale, Oslo Social Support Scale-3, Perceived Relationship Quality Components Scale, Experiences in Close Relationships Scale-Revised, Talking to Strangers Scale). The full text of these metrics are provided on this paper’s OSF page along with a detailed description of how they are being utilized for separate projects/papers.
